# Uniparental maternal tetrasomy X co-occurrence with paternal nondisjunction: investigation of the origin of 48,XXXX

**DOI:** 10.1038/s41439-024-00289-6

**Published:** 2024-08-16

**Authors:** Keiko Shimojima Yamamoto, Sakurako Yamamoto, Taichi Imaizumi, Satoko Kumada, Toshiyuki Yamamoto

**Affiliations:** 1 Department of Transfusion Medicine and Cell Processing, Tokyo Women’s Medical University, Tokyo, 162-8666 Japan; 2 Institute of Medical Genetics, Tokyo Women’s Medical University, Tokyo, 162-8666 Japan; 3 Department of Neuropediatrics, Tokyo Metropolitan Neurological Hospital, Fuchu, 183-0042 Japan; 4 Department of Pediatrics, St. Marianna University School of Medicine, Kawasaki, 216-8511 Japan; 5 Division of Gene Medicine, Graduate School of Medicine, Tokyo Women’s Medical University, Tokyo, 162-8666 Japan

**Keywords:** Cytogenetics, Genetics research

## Abstract

Tetrasomy X or 48,XXXX is a rare sex chromosome aneuploidy. The parental origin of tetrasomy X in a female patient with developmental delay was analyzed; all four X chromosomes were derived from the mother, and there were no paternally derived sex chromosomes. This finding indicates a rare incidental co-occurrence of maternal and paternal nondisjunction or polysomy rescue. The mechanism of 48,XXYY, which is related to developmental delay in males, was analyzed for comparison.

Sex chromosome aneuploidies such as 45,X, 47,XXX, and 47,XXY are common. However, sex chromosome aneuploidies involving more than four sex chromosomes are rare. Although three sex chromosome aneuploidies are rarely related to developmental delay, patients with more than four sex chromosome aneuploidies often experience developmental delays of variable severity^1–3^. In mice, maternally inherited X chromosomes fail to be inactivated in the embryo, which reflects the well-established imprinting of mouse X chromosomes^4^. However, X-chromosome inactivation (XCI) in human embryos is not subject to the same imprinting influence that is observed in mice, indicating that there is no parental origin-specific epigenetic modification in the human X chromosome. Alternatively, random X inactivation in XX females compensates for the dosage difference of X chromosomes compared with XY males^5^. In polysomy X, all but one (n-1) X chromosomes are inactivated. However, in polysomy X, XCI may be incomplete, and the dosage sensitivity of genes that escape XCI may contribute to the phenotype^6–8^.

Tetrasomy X or 48,XXXX is a rare sex chromosome aneuploidy that was first reported in 1961^9^. To date, more than 50 cases have been reported, including associations with several clinical features, such as developmental delay and premature ovarian failure^3,10,11^.

Recently, we encountered a girl with moderate developmental delay in whom a rare sex chromosome aberration, tetrasomy X, was identified. To better understand this mechanism, we analyzed the origins of the four X chromosomes. For comparison, we also analyzed the mechanism of 48,XXYY, which is related to developmental delay in males.

This study was performed according to the Declaration of Helsinki, and the requisite permission was obtained from the institutional ethics committee. This study enrolled patients whose sex chromosome aneuploidies were detected by conventional chromosomal analysis. To further evaluate trio samples, including the patients and their parents, comparative genomic hybridization (CGH) associated with single-nucleotide polymorphism (SNP) analysis was performed using an Agilent CGH + SNP 180 K array (Agilent Technologies, Santa Clara, CA) as described previously^12^. Peripheral blood samples were collected after written informed consent was obtained from patients and their parents. For the reference samples, sex-matched control DNAs purchased from Agilent Technologies were used: “European Female” for Patient 1 and “European Male” for Patient 2. Fluorescence in situ hybridization (FISH) was performed to confirm the existence of additional sex chromosomes, as described previously^13^. Bacterial artificial chromosome clones of the X chromosome, RP11-75D20 (Xp22.13) and RP11-244I10 (Xq28), were labeled with Spectrum Orange and Green, respectively, and used as FISH probes. For the Y chromosome, RP11-203A18 (Yq11.221) and CTD-2291B20 (Yq11.23) were labeled with Spectrum Orange and Green, respectively. To confirm the parental origin of the X chromosomes, inherited patterns of the haplotype within the families were checked against the results of the SNP types of the patients and their parents, which were extracted using Agilent CytoGenomics software (Agilent Technologies). Furthermore, microsatellite marker analysis was performed for the family of Patient 1 using the ABI Prism Linkage Mapping Set with DXS990 and analyzed using the SeqStudio Genetic Analyzer and GeneMapper software (Thermo Fisher Scientific, Waltham, MA).

Patient 1 (a 3-year-old girl) was born at 41 weeks and 2 days of gestation. The patient’s father and mother were 36 and 31 years old at delivery, respectively. This patient was the only child of her parents. There was no history of miscarriage. The patient’s family history was unremarkable. The parents were not consanguineous. The patient’s birth weight was 2850 g (25th–50th centile), the length was 50 cm (75th–90th centile), and the occipitofrontal circumference (OFC) was 34.5 cm (75th–90th centile). Gross motor development was mildly delayed, with head control achieved at 4.5 months, rolling over at 10 months, sitting at 11 months, standing with support at 18 months, and walking alone at 24 months. Language development was also delayed; meaningful words started at 11 months, but there were no two-word sentences at three years. At present, her height is 91.5 cm (25th–50th centile), her weight is 13.3 kg (50th–75th centile), and her OFC is 47 cm (10th–25th centile). Her facial features were distinctive, with hypertelorism, a flat nasal bridge, and micrognathia. Physical examination of the oral, chest, and abdominal areas revealed no abnormalities; however, a neurological examination revealed mild hypotonia. Specifically, she exhibited an expanded range of motion of the elbow and shoulder joints. Flat feet were also observed. The patient demonstrated wide-based walking and was unable to jump. Eye contact was good, but intermittent exotropia was often observed. She could not put on or remove clothes. Toilet habits had not yet been established. She could identify color names but could not distinguish differences in size and length. Brain magnetic resonance imaging performed during infancy revealed no abnormalities. Conventional chromosomal microarray testing was performed at 30 months of age. The results revealed tetrasomy X and no other pathogenic copy number aberrations.

In Patient 1, CGH revealed a quadruplication pattern (log_2_ ratio≒1) through the X chromosome (Fig. 1A). The loss-of-heterozygosity (LOH) analysis revealed the existence of four alleles but only two haplotypes (Fig. 1B), which is extremely rare. FISH analysis confirmed the existence of four X chromosomes (Fig. 2A). X chromosome SNP typing patterns were obtained using CGH + SNP analysis and are presented in Supplemental Table S1. Approximately half of the SNP data were noninformative owing to the lack of at least one SNP type among the three sets of data for Patient 1 and her parents. However, many SNPs were found to be derived solely from the mother. Microsatellite marker analysis was performed, and Patient 1 presented only two peaks that were common to her mother (Fig. 3), which indicates that Patient 1 inherited two haplotypes from her mother. Furthermore, the peak ratio was the same as that of her mother. Thus, the two maternally derived X chromosomes were confirmed to be equally duplicated.

**Fig. 1:**
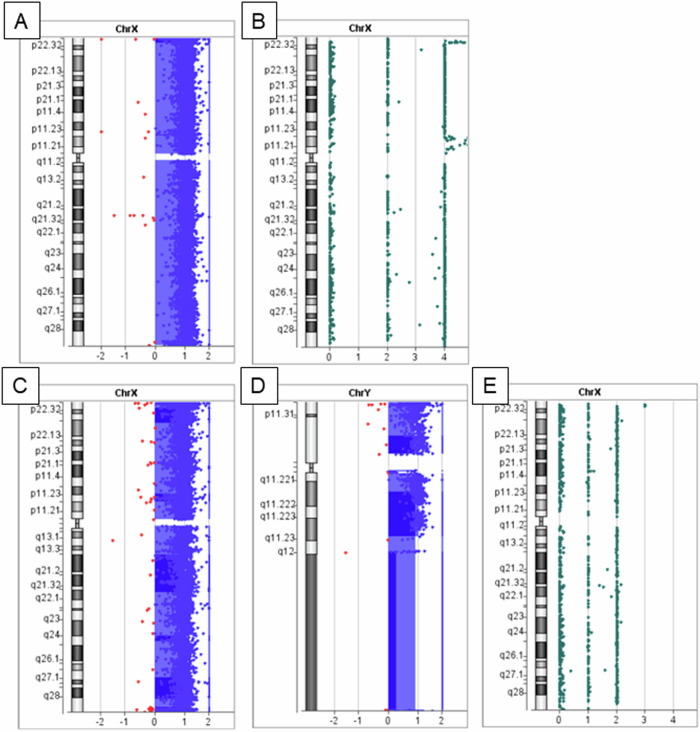
Results of CGH + SNP analysis for sex chromosomes. CGH analyses (**A**, **C**, and **D**) and LOH analyses (**B**, **E**) for Patient 1 (**A**, **B**) and Patient 2 (**C**, **D**, and **E**) are shown for the X chromosome (**A**, **B**, **C**, and **E**) and Y chromosome (**D**). All CGH views demonstrate a gain of log_2_ ratio≒1. Because a female control sample is used for Patient 1, a log_2_ ratio≒1 indicates tetrasomy. For Patient 2, a male control sample is used, and a log_2_ ratio≒1 indicates disomy. The LOH view for Patient 1 (**B**) demonstrated the existence of four X chromosomes but only two haplotypes. The LOH view for Patient 2 (**E**) shows the existence of two X chromosomes with heterozygosity.

**Fig. 2:**
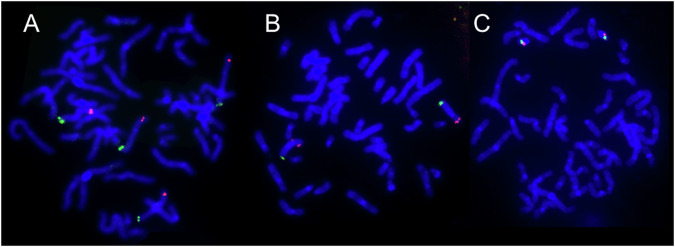
Results of FISH analysis. For Patient 1, the existence of four independent X chromosomes is shown (**A**). For Patient 2, two X chromosomes (**B**) and two Y chromosomes (**C**) are shown.

**Fig. 3:**
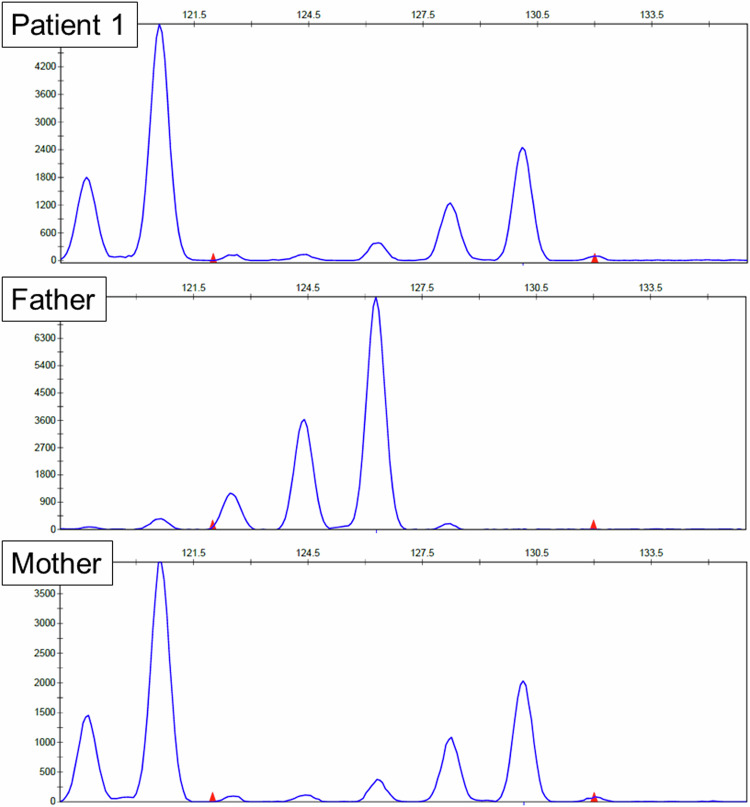
Results of microsatellite marker analysis. Patient 1 shows two peaks in common with her mother but no peak in common with her father. The patient’s peak height ratio is the same as that of her mother, indicating that they have the same copy number.

The LOH analysis of Patient 1 showed that there were only two haplotypes among the four X chromosomes, and this pattern cannot be explained by nondisjunction in one of the parents. Patient 1 had four X chromosomes and two haplotypes common to the patient’s mother. The two maternal X chromosomes were duplicated equally. Thus, successive nondisjunction occurred during meiosis I and II in oocytes (Fig. 4A). However, this result does not explain the absence of paternally derived sex chromosomes. Therefore, the incidental occurrence of nondisjunction of sex chromosomes during paternal meiosis may be considered an additional mechanism.

**Fig. 4:**
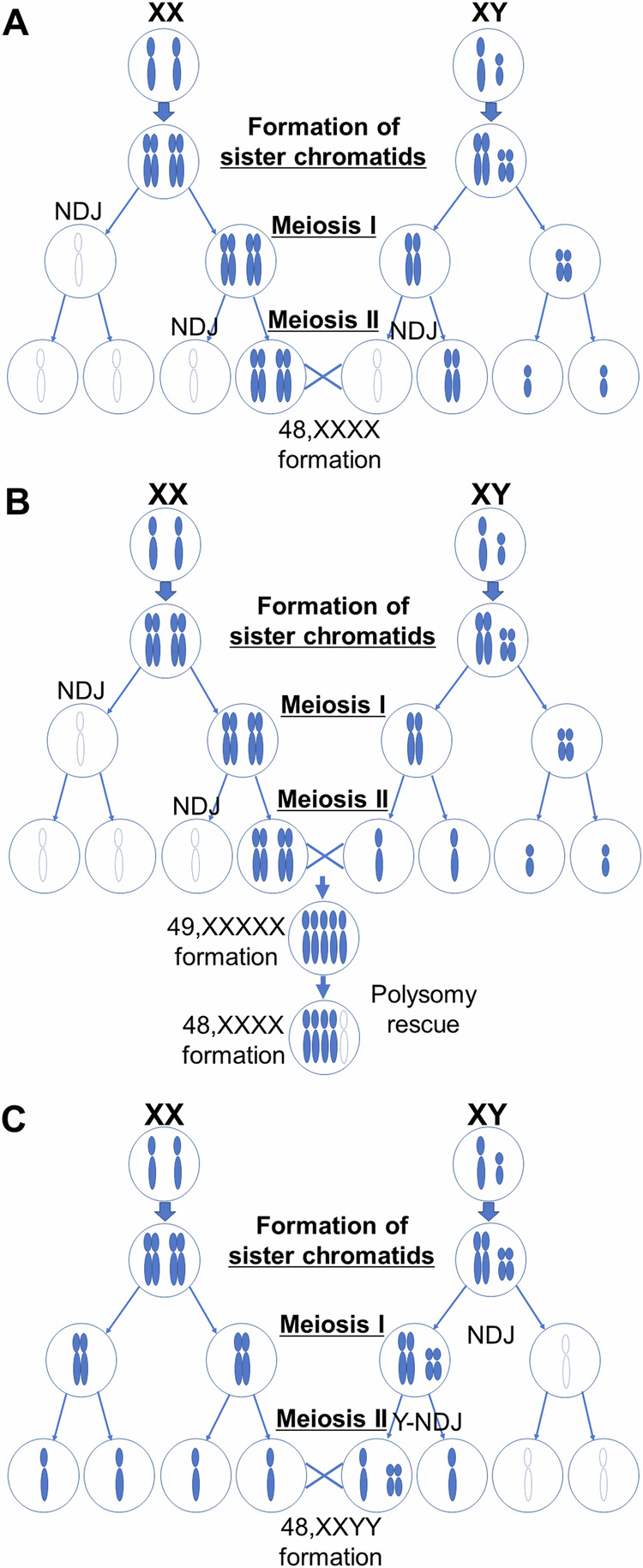
Suspected mechanism of sex chromosome polysomy in this study. NDJ indicates “nondisjunction”. In Patient 1 (**A**), successive NDJ occurrs during meiosis of the oocyte, accompanying paternal NDJ. An alternative mechanism (**B**) is possible polysomy rescue after the formation of 49,XXXXX. In Patient 2 (**C**), successive NDJ occurrs in the paternally derived allele. Y-NDJ means that the second NDJ occurs only on the Y chromosome.

Many reports have analyzed the parental origins of extra sex chromosomes in patients with X chromosome polysomy^14–24^. Most previous reports have shown that most extra-sex chromosomes are maternally derived with one paternally derived sex chromosome (X or Y chromosome). This finding indicated successive nondisjunction of the X chromosomes during meiosis I and II in the oocytes. Thus, 49,XXXXX and 49,XXXXY are equivalent^25^. In these two patterns, paternal nondisjunction was not related to the results.

With respect to 48,XXXX, three mechanisms were considered. First, three X chromosomes were of maternal origin, and the fourth X chromosome was of paternal origin. In this pattern, nondisjunction of the X chromosome occurs during meiosis I or II in oocytes. Most cases of 48,XXXX are derived through this mechanism. Second, all four X chromosomes were of maternal origin with successive nondisjunction in meiosis I and II, and no sex chromosomes were of paternal origin because the nondisjunction of sex chromosomes occurred by chance (Fig. 4A). Therefore, the incidental co-occurrence of maternally and paternally derived nondisjunction is extremely rare. The third mechanism is possible polysomy rescue (Fig. 4B), similar to the system for removing an extra chromosome known as trisomy rescue; one of the X chromosomes of 49,XXXXX or the Y chromosome of 49,XXXXY may be lost during embryogenesis^26^. Although the existence of this mechanism has never been proven, the second or third mechanisms were considered for Patient 1 in this study. To the best of our knowledge, only two patients in whom uniparental maternal tetrasomy X was confirmed have been reported previously^27,28^.

There is a clear haplotype difference between the two types of 48,XXXX, those with three X chromosomes originating from the mother and those with all four X chromosomes originating from the mother. These patterns can be easily distinguished using CGH + SNP analysis. Only the second and third mechanisms would show two haplotype patterns, as observed in Patient 1 in this study, which can indicate uniparental tetrasomy. Attention should be given to the LOH analysis to differentiate them.

Patient 2 (an additional patient) was a 3-year-old boy. The patient was born at 38 weeks and 4 days of gestation. His birth weight was 2665 g (10th–25th centile), his length was 47.0 cm (10th–25th centile), and his OFC was 30.3 cm (< 3rd centile). His parents were healthy and nonconsanguineous. His father and mother were 37 and 35 years old, respectively. He had two healthy older sisters. Since infancy, he has exhibited psychomotor developmental delay, generalized hypotonia, left polycystic kidney, internal curvature of the fifth finger on both sides, distinctive features of the epicanthus, narrow philtrum, and fused mandibular teeth. On the basis of these findings, conventional G-band chromosomal analysis was performed, and an abnormal karyotype of 48,XXYY was identified. At present, his height is 90.8 cm (10th–25th centile), his weight is 12.3 kg (10th–25th centile), and his OFC is 49.0 cm (25th–50th centile).

In Patient 2, CGH revealed a diploid pattern of X- and Y-chromosomes (log_2_ ratio≒1) (Fig. 1C, D). The LOH analysis revealed a heterozygous pattern of the X chromosome (Fig. 1E). This finding is also commonly observed in healthy females. For the Y chromosome, the SNP was not included on the platform. Thus, a duplication of the Y chromosome was detected, but the LOH pattern could not be confirmed. Two X chromosomes (Fig. 2B) and two Y chromosomes (Fig. 2C) were confirmed by FISH analysis. Most SNP types on the X chromosomes were common to both the father and mother (Supplemental Table S2). Two X chromosomes were inherited from each parent. However, the two Y chromosomes were definitely inherited from his father. Because no SNP probes are designed for the Y chromosome, it is unknown whether LOH exists between the two Y chromosomes. However, it can be speculated that nondisjunction of sex chromosomes (X- and Y-chromosomes) occurred during paternal meiosis I, and successive nondisjunction of Y chromosomes occurred during meiosis II (Fig. 4C). This mechanism could explain the three sex chromosomes inherited from the father. This finding was similar to that previously reported in patients.

In this study, Patient 1 with 48,XXXX presented mild developmental delay. Patient 2 with 48,XXYY also exhibited developmental delay. There is no contradiction in the relationship between chromosomal abnormality patterns and clinical symptoms^2,3,29^. Even considering the lack of correlation between advanced maternal age and an increased risk of polysomy X^30^, the parents of both patients in this study were not of advanced maternal age, and there was no contradiction.

## Supplementary information

Supplemental Table S2

Supplemental Table S1

## Data Availability

The data that support the findings of this study are available from the corresponding author upon reasonable request.
