# Supplementary material for: Uniparental maternal tetrasomy X co-occurrence with paternal nondisjunction: investigation of the origin of 48,XXXX
Source: Hum Genome Var. 2024 Aug 16;11:31. doi: 10.1038/s41439-024-00289-6 (PMC11329761; doi:10.1038/s41439-024-00289-6)
Supplement: Supplementary file 2 — Supplemental Table S1 [file 41439_2024_289_MOESM2_ESM.pdf]

Supplemental Table S1. SNP typing of the patient 1 family

SNP ID	Probe Name	Chromosome	SNP Position	Feature Number	Father	Mother	Proband
rs7879691	A_20_P00300756	chrX	492888	33667	GG	TG	TT
rs7061452	A_20_P00300759	chrX	653664	63238	NN	NN	NN
rs6644250	A_20_P00303799	chrX	770203	69741	NN	NN	NN
rs5988407	A_20_P00198855	chrX	782630	31955	NN	NN	NN
rs5946520	A_20_P00300762	chrX	827550	171644	NN	GT	GG
rs5989939	A_20_P00300767	chrX	1550779	21121	GC	GC	GG
rs5948931	A_20_P00300769	chrX	1632939	98181	NN	NN	NN
rs6644727	A_20_P00201895	chrX	1640371	46872	NN	NN	NN
rs5948969	A_20_P00300770	chrX	1681017	56229	NN	TT	TT
rs6588799	A_20_P00201896	chrX	1702957	83260	NN	GC	GG
rs7887304	A_20_P00201897	chrX	1805618	126979	NN	NN	NN
rs7883731	A_20_P00300774	chrX	1904088	32358	GG	GG	GG
rs7056197	A_20_P00300775	chrX	1952600	113620	NN	NN	NN
rs5939175	A_20_P00201899	chrX	2326782	23594	NN	CG	CC
rs5982769	A_20_P00300777	chrX	2334418	63264	GG	GG	GG
rs5939093	A_20_P00300778	chrX	2339117	9612	GG	GG	GG
rs1394772	A_20_P00300780	chrX	2466724	138597	TT	TT	TT
rs17842888	A_20_P00198875	chrX	2476073	167030	GC	GG	GG
rs17842897	A_20_P00300782	chrX	2480894	142803	TT	TT	TT
rs5939262	A_20_P00198877	chrX	2562338	21578	NN	CC	CC
rs7878926	A_20_P00201903	chrX	2589272	27900	NN	NN	NN
rs1700949	A_20_P00198879	chrX	2590115	104876	TT	TT	TT
rs17842915	A_20_P00300786	chrX	2595450	94629	NN	NN	NN
rs311058	A_20_P00300788	chrX	2626860	163870	CC	CC	CC
rs311154	A_20_P00300791	chrX	2690827	98741	CG	CC	CC
rs311184	A_20_P00300792	chrX	2714965	57267	NN	NN	GGGG
rs5939382	A_20_P00198887	chrX	2787455	54009	A	AG	AAGG
rs5982603	A_20_P00198888	chrX	2788707	140019	NN	TC	CCCC
rs5939139	A_20_P00300795	chrX	2791081	158950	T	CC	CCCC
rs2694731	A_20_P00198891	chrX	3129641	127327	NN	NN	NN
rs17051542	A_20_P00300799	chrX	3137450	65122	C	TC	TTCC
rs1635212	A_20_P00198896	chrX	3216117	163897	G	GG	GGGG
rs806620	A_20_P00300806	chrX	3279941	33369	G	NN	NN
rs1624449	A_20_P00198903	chrX	3293025	110422	A	AG	AAGG
rs5982706	A_20_P00198905	chrX	3314094	36139	NN	NN	NN
rs12398525	A_20_P00300815	chrX	3371825	176018	C	NN	NN
rs6567526	A_20_P00300816	chrX	3381343	143618	C	NN	NN
rs7053894	A_20_P00198911	chrX	3386599	63626	C	CC	CCCC
rs12392003	A_20_P00198912	chrX	3394743	102466	NN	NN	NN
rs7473099	A_20_P00300819	chrX	3435609	119989	T	TC	TTCC
rs6641786	A_20_P00300820	chrX	3447762	23729	T	CT	CCTT
rs6641787	A_20_P00300821	chrX	3447922	170260	NN	CT	CCTT
rs10747229	A_20_P00198916	chrX	3502739	44586	NN	NN	NN
rs3848932	A_20_P00300824	chrX	3942684	48057	T	TT	TTTT
rs5915686	A_20_P00300826	chrX	3969879	48549	NN	NN	NN
rs4273976	A_20_P00198921	chrX	3978015	153356	G	GG	GGGG

SNP ID	Probe Name	Chromosome	SNP Position	Feature Number	Father	Mother	Proband
rs5916443	A_20_P00300828	chrX	3979702	176930	G	AG	AAGG
rs4449947	A_20_P00198923	chrX	3982858	39122	A	AA	AAAA
rs5916465	A_20_P00300831	chrX	4016482	9592	A	AA	AAAA
rs5916468	A_20_P00300832	chrX	4034173	5164	T	TT	TTTT
rs6639309	A_20_P00300833	chrX	4045114	128220	T	NN	NN
rs5961458	A_20_P00198928	chrX	4047293	100448	NN	TA	TTAA
rs6529803	A_20_P00198930	chrX	4083547	89824	C	CC	CCCC
rs4826875	A_20_P00198931	chrX	4116652	142106	C	TC	TTCC
rs5962095	A_20_P00198932	chrX	4119860	82666	C	TT	TTTT
rs5916549	A_20_P00300840	chrX	4159803	138254	T	TT	TTTT
rs12846730	A_20_P00300841	chrX	4159945	46806	G	GG	GGGG
rs5916554	A_20_P00198936	chrX	4160830	63384	NN	NN	NN
rs12846672	A_20_P00300843	chrX	4174298	87033	G	GG	GGGG
rs5961484	A_20_P00300844	chrX	4197200	143689	G	GG	GGGG
rs5915751	A_20_P00300848	chrX	4419706	132477	NN	AA	AAAA
rs6640263	A_20_P00198943	chrX	4426159	10424	NN	NN	NN
rs6640309	A_20_P00300850	chrX	4449765	48827	C	CC	NN
rs7061816	A_20_P00198947	chrX	4643018	72450	T	CT	CCTT
rs17313910	A_20_P00300854	chrX	4671915	163519	C	TC	TTCC
rs7882203	A_20_P00198949	chrX	4714113	44099	G	GG	NN
rs7890840	A_20_P00198950	chrX	4715468	154933	G	GG	GGGG
rs17267288	A_20_P00300857	chrX	4720073	83663	NN	CC	CCCC
rs7886221	A_20_P00198952	chrX	4850704	94599	NN	NN	NN
rs17219378	A_20_P00198953	chrX	4871591	106709	C	CC	CCCC
rs5915462	A_20_P00198955	chrX	4896439	39306	NN	GG	GGGG
rs6639258	A_20_P00300863	chrX	4955230	151235	G	AA	AAAA
rs5915926	A_20_P00198958	chrX	4979267	33777	G	GG	GGGG
rs1900768	A_20_P00300866	chrX	5023991	120758	G	AA	AAAA
rs1462636	A_20_P00198961	chrX	5045078	46128	NN	AA	AAAA
rs6639312	A_20_P00300868	chrX	5051154	115222	G	AA	AAAA
rs5961627	A_20_P00300869	chrX	5052447	91509	G	AA	AAAA
rs6639398	A_20_P00198965	chrX	5141299	92983	NN	AA	AAAA
rs12011810	A_20_P00300873	chrX	5308092	173698	T	TT	TTTT
rs12012674	A_20_P00198968	chrX	5330377	55702	C	NN	NN
rs5961771	A_20_P00300879	chrX	5560788	87117	C	CC	CCCC
rs12394831	A_20_P00300880	chrX	5563321	132711	A	AA	AAAA
rs5916197	A_20_P00198975	chrX	5590016	40223	G	GG	GGGG
rs6639485	A_20_P00198977	chrX	5660184	37298	C	CC	CCCC
rs1842835	A_20_P00198978	chrX	5660396	136404	NN	GG	GGGG
rs5961840	A_20_P00300886	chrX	5702122	83332	C	CC	CCCC
rs5961854	A_20_P00198981	chrX	5717394	152570	A	AA	AAAA
rs5961367	A_20_P00300888	chrX	5724406	74873	NN	TT	TTTT
rs5961868	A_20_P00198983	chrX	5740117	12163	G	NN	NN
rs17305726	A_20_P00300890	chrX	5770271	163645	C	CC	CCCC
rs16997473	A_20_P00198985	chrX	5786486	88606	G	GG	NN
rs5916269	A_20_P00198986	chrX	5810299	173794	G	NN	NN
rs4358933	A_20_P00300893	chrX	5815502	137541	C	CG	CCGG

SNP ID	Probe Name	Chromosome	SNP Position	Feature Number	Father	Mother	Proband
rs2369486	A_20_P00198988	chrX	5865656	123470	C	CC	CCCC
rs6639547	A_20_P00198989	chrX	5877510	179973	C	CC	CCCC
rs12012019	A_20_P00198991	chrX	5919376	34719	NN	NN	NN
rs10522048	A_20_P00300899	chrX	5999991	121276	C	CG	CCGG
rs1921943	A_20_P00198994	chrX	6013758	29424	T	TT	TTTT
rs11798753	A_20_P00198995	chrX	6024351	76791	A	CC	CCCC
rs5916352	A_20_P00300905	chrX	6142368	126192	NN	CC	CCCC
rs7050121	A_20_P00199000	chrX	6150982	8341	NN	CC	NN
rs5916359	A_20_P00300907	chrX	6158061	23297	T	TC	TTTC
rs4826724	A_20_P00300909	chrX	6262277	102489	NN	CC	CCCC
rs2011676	A_20_P00199004	chrX	6322001	49536	NN	NN	NN
rs12395835	A_20_P00199006	chrX	6350397	63652	C	NN	NN
rs1921513	A_20_P00199007	chrX	6351305	83389	G	GG	GGGG
rs7064241	A_20_P00300914	chrX	6359723	75655	G	GG	GGGG
rs1921522	A_20_P00300915	chrX	6366114	157654	NN	NN	NN
rs7062858	A_20_P00199011	chrX	6376879	103270	A	AA	AAAA
rs16984199	A_20_P00199012	chrX	6435309	103647	G	NN	NN
rs5948735	A_20_P00300919	chrX	6482092	61217	NN	NN	NN
rs7888073	A_20_P00199014	chrX	6494230	102220	T	TT	TTTT
rs1450152	A_20_P00300921	chrX	6580181	86927	NN	AA	AAAA
rs4914906	A_20_P00300923	chrX	6668181	127959	NN	GG	GGGG
rs11095297	A_20_P00199020	chrX	6904445	85187	NN	GG	GGGG
rs7050156	A_20_P00199021	chrX	6997528	74278	A	AA	AAAA
rs7057654	A_20_P00199022	chrX	7002837	149872	NN	GG	GGGG
rs1465067	A_20_P00300929	chrX	7007959	39271	C	AA	AAAA
rs2293434	A_20_P00199024	chrX	7072831	170747	G	AA	AAAA
rs802913	A_20_P00300932	chrX	7108850	163173	T	TT	TTTT
rs10521600	A_20_P00199028	chrX	7269320	32446	A	AA	AAAA
rs6530079	A_20_P00300935	chrX	7277081	25053	C	NN	NN
rs7884548	A_20_P00199030	chrX	7318293	170421	NN	AA	NN
rs16984495	A_20_P00199031	chrX	7328958	117596	NN	GG	GGGG
rs7879965	A_20_P00199032	chrX	7383909	22281	C	CC	NN
rs7056055	A_20_P00199033	chrX	7514402	31355	NN	CC	NN
rs6639897	A_20_P00199034	chrX	7533100	95405	G	GC	GGCC
rs12010606	A_20_P00199036	chrX	7560345	117584	NN	NN	NN
rs923644	A_20_P00199037	chrX	7561263	132124	NN	NN	NN
rs16984614	A_20_P00300946	chrX	7606325	122631	C	AC	AACC
rs12687154	A_20_P00199041	chrX	7614386	73829	NN	CC	CCCC
rs7055344	A_20_P00199042	chrX	7615046	147175	C	NN	CCCC
rs7064675	A_20_P00199043	chrX	7681601	57989	NN	TT	TTTT
rs16984660	A_20_P00300950	chrX	7685731	68625	NN	NN	NN
rs6530118	A_20_P00199048	chrX	7822751	95741	NN	CC	CCCC
rs5980259	A_20_P00199049	chrX	7844837	105985	A	GA	GGAA
rs1179135	A_20_P00199050	chrX	7884996	119327	T	NN	NN
rs16984818	A_20_P00199051	chrX	7932124	125106	G	GG	GGGG
rs5978803	A_20_P00300958	chrX	7961755	35212	NN	NN	NN
rs5978805	A_20_P00199053	chrX	7972181	117545	NN	NN	TTTT

SNP ID	Probe Name	Chromosome	SNP Position	Feature Number	Father	Mother	Proband
rs5934336	A_20_P00300960	chrX	7988544	177906	G	NN	GGGG
rs5934376	A_20_P00199056	chrX	8053206	13704	C	CC	CCCC
rs11095480	A_20_P00300963	chrX	8074571	132057	G	TT	TTTT
rs7056974	A_20_P00199058	chrX	8115453	134405	NN	NN	NN
rs7885180	A_20_P00300965	chrX	8125314	6130	A	AA	AAAA
rs12557767	A_20_P00199060	chrX	8177514	112982	NN	GG	GGGG
rs17306621	A_20_P00300969	chrX	8347615	139814	C	TC	TTCC
rs4830581	A_20_P00199064	chrX	8355560	98154	NN	NN	NN
rs6638794	A_20_P00300971	chrX	8371913	111942	NN	NN	NN
rs7892812	A_20_P00300972	chrX	8394253	109385	G	NN	NN
rs1639119	A_20_P00199069	chrX	8508062	167555	T	TT	TTTT
rs5933667	A_20_P00300976	chrX	8535439	167409	C	CC	CCCC
rs7877867	A_20_P00300979	chrX	8595303	88871	A	NN	NN
rs6654877	A_20_P00300980	chrX	8599261	30542	G	NN	NN
rs12008923	A_20_P00300981	chrX	8621660	26557	C	CC	CCCC
rs6530187	A_20_P00300983	chrX	8646483	137572	T	TC	TTCC
rs7063364	A_20_P00199079	chrX	8757347	142480	NN	NN	NN
rs1985209	A_20_P00300986	chrX	8787078	70815	A	AA	AAAA
rs1997347	A_20_P00300987	chrX	8926779	39415	C	CC	CCCC
rs5978285	A_20_P00199082	chrX	8962312	123804	NN	TT	TTTT
rs6640319	A_20_P00300989	chrX	9199178	47457	T	CT	CCTT
rs6530269	A_20_P00199086	chrX	9335171	6248	NN	GA	GGAA
rs4595306	A_20_P00300993	chrX	9418597	167655	NN	GG	GGGG
rs756949	A_20_P00199092	chrX	9531161	1103	G	TT	TTTT
rs2015165	A_20_P00199093	chrX	9548193	56057	NN	NN	NN
rs5979162	A_20_P00199100	chrX	9696507	80273	A	NN	NN
rs5934683	A_20_P00301007	chrX	9751474	40595	NN	TT	TTTT
rs13440489	A_20_P00301008	chrX	9768342	49067	A	NN	NN
rs7051394	A_20_P00301009	chrX	9786503	127469	NN	NN	NN
rs757020	A_20_P00199104	chrX	9825088	2640	G	GG	GGGG
rs17327510	A_20_P00199105	chrX	9846539	146240	G	TT	TTTT
rs6640537	A_20_P00301012	chrX	9847717	171739	NN	NN	NN
rs1003826	A_20_P00301015	chrX	9896893	134854	G	NN	NN
rs2188385	A_20_P00301018	chrX	9923646	173774	G	AA	AAAA
rs12842066	A_20_P00199114	chrX	10036798	9014	G	GG	GGGG
rs2285492	A_20_P00199115	chrX	10066949	96608	NN	AT	AATT
rs17255432	A_20_P00301025	chrX	10182560	110546	G	NN	NN
rs1007461	A_20_P00301027	chrX	10297210	66454	A	AA	AAAA
rs5934857	A_20_P00199122	chrX	10299297	93699	NN	NN	NN
rs5933845	A_20_P00301029	chrX	10355280	131230	NN	GA	GGAA
rs16986120	A_20_P00199124	chrX	10368605	118995	NN	NN	NN
rs2216426	A_20_P00199127	chrX	10463103	166212	C	TT	TTTT
rs6530404	A_20_P00301034	chrX	10541670	100775	T	NN	NN
rs10521612	A_20_P00301035	chrX	10585521	152683	G	GG	NN
rs7059850	A_20_P00301036	chrX	10632780	154516	NN	TT	TTTT
rs5979340	A_20_P00199131	chrX	10655565	52877	T	TT	TTTT
rs12009787	A_20_P00301038	chrX	10799244	33471	NN	NN	NN

SNP ID	Probe Name	Chromosome	SNP Position	Feature Number	Father	Mother	Proband
rs41471549	A_20_P00301039	chrX	11155336	54330	NN	NN	NN
rs2057652	A_20_P00199134	chrX	11167681	157253	A	AA	NN
rs5934967	A_20_P00301042	chrX	11174565	50222	NN	NN	CCCC
rs6640718	A_20_P00301043	chrX	11211981	56609	NN	AA	AAAA
rs5935008	A_20_P00301044	chrX	11350610	54853	C	NN	NN
rs5933878	A_20_P00301045	chrX	11373487	173695	NN	NN	NN
rs6639071	A_20_P00301048	chrX	11672462	16436	G	NN	NN
rs5935142	A_20_P00301049	chrX	11722572	155128	NN	GG	GGGG
rs6639075	A_20_P00301050	chrX	11738819	123787	G	GG	GGGG
rs2023901	A_20_P00199145	chrX	11750255	47211	G	GG	GGGG
rs886481	A_20_P00199147	chrX	11806458	7370	A	AA	AAAA
rs7877805	A_20_P00301054	chrX	11814380	112529	C	NN	CCCC
rs858083	A_20_P00301055	chrX	11821187	152189	G	NN	GGGG
rs5979482	A_20_P00301056	chrX	11878254	83644	C	NN	NN
rs6639121	A_20_P00199153	chrX	12017668	111370	G	CC	CCCC
rs12859698	A_20_P00199154	chrX	12187836	31091	NN	CC	CCCC
rs5978493	A_20_P00301061	chrX	12191372	107799	C	CC	CCCC
rs12690196	A_20_P00301062	chrX	12201083	51501	G	NN	NN
rs5933969	A_20_P00199157	chrX	12225249	150607	T	TT	TTTT
rs7888255	A_20_P00301064	chrX	12274667	64633	NN	CC	CCCC
rs13440707	A_20_P00301065	chrX	12313140	93094	C	CC	CCCC
rs5935297	A_20_P00301066	chrX	12343389	51593	NN	AA	AAAA
rs6530506	A_20_P00199161	chrX	12358893	136095	T	TT	TTTT
rs1316790	A_20_P00199162	chrX	12395376	15228	NN	NN	NN
rs5935327	A_20_P00301069	chrX	12420503	23306	NN	CC	CCCC
rs1317151	A_20_P00301070	chrX	12489590	61466	G	GG	GGGG
rs721047	A_20_P00199165	chrX	12502695	13709	C	NN	NN
rs5979639	A_20_P00199166	chrX	12518481	129530	NN	NN	NN
rs6641055	A_20_P00199168	chrX	12587037	175449	G	NN	NN
rs5934030	A_20_P00199173	chrX	12719749	50166	NN	AA	AAAA
rs4830797	A_20_P00199174	chrX	12723615	17018	T	CC	CCCC
rs7880793	A_20_P00199175	chrX	12725464	52568	T	TT	NN
rs1268602	A_20_P00301083	chrX	12795821	164765	A	AC	AACC
rs4639691	A_20_P00301085	chrX	12824758	11322	NN	GT	GGTT
rs16987131	A_20_P00301087	chrX	12862722	42255	A	AA	AAAA
rs5744067	A_20_P00301089	chrX	12934973	127369	C	NN	NN
rs5979798	A_20_P00199186	chrX	13058127	151113	C	CC	NN
rs5935502	A_20_P00199187	chrX	13111858	109567	NN	GT	GGTT
rs5978614	A_20_P00199189	chrX	13248625	97939	A	AA	NN
rs12862147	A_20_P00199190	chrX	13253147	47233	T	TT	TTTT
rs917014	A_20_P00301097	chrX	13363731	179805	NN	TT	TTTT
rs17322192	A_20_P00199192	chrX	13418107	92395	NN	CG	CCGG
rs5935585	A_20_P00301101	chrX	13518104	65827	NN	NN	NN
rs6527839	A_20_P00199197	chrX	13592881	180741	NN	NN	NN
rs5935616	A_20_P00199198	chrX	13596261	135379	NN	NN	NN
rs16979108	A_20_P00301107	chrX	13702705	138436	C	CC	CCCC
rs7066605	A_20_P00301108	chrX	13849695	113288	G	GG	GGGG

SNP ID	Probe Name	Chromosome	SNP Position	Feature Number	Father	Mother	Proband
rs2188768	A_20_P00301109	chrX	13894378	145537	C	CC	CCCC
rs6633417	A_20_P00199204	chrX	13930495	14076	NN	GG	GGGG
rs6528024	A_20_P00301111	chrX	13930922	159251	C	TT	TTTT
rs2285632	A_20_P00301113	chrX	14010282	134530	NN	CC	CCCC
rs1557726	A_20_P00199208	chrX	14021807	101452	C	CC	CCCC
rs12006779	A_20_P00199209	chrX	14058661	72493	T	TT	TTTT
rs16979405	A_20_P00199210	chrX	14095546	171403	A	GG	GGGG
rs7049464	A_20_P00301117	chrX	14288509	175228	G	NN	NN
rs7885870	A_20_P00301119	chrX	14311238	135138	NN	NN	NN
rs6628196	A_20_P00301121	chrX	14427630	137076	C	CC	CCCC
rs7060100	A_20_P00199216	chrX	14429311	20977	A	AA	AAAA
rs7879330	A_20_P00199217	chrX	14485645	107719	G	GG	GGGG
rs4565623	A_20_P00301124	chrX	14523772	95892	G	GG	GGGG
rs4366225	A_20_P00199219	chrX	14529475	23813	G	GG	NN
rs3027322	A_20_P00199220	chrX	14553073	115385	C	NN	CCCC
rs5935789	A_20_P00301132	chrX	14698983	35014	T	TC	TTTT
rs5935795	A_20_P00301133	chrX	14717421	95438	NN	NN	AAAA
rs3922640	A_20_P00199228	chrX	14791296	48044	A	NN	AAAA
rs5935812	A_20_P00301135	chrX	14859655	162218	NN	CC	CCCC
rs2905220	A_20_P00301136	chrX	14864003	108916	NN	CC	CCCC
rs5980081	A_20_P00199232	chrX	14979361	75812	C	CC	CCCC
rs5980082	A_20_P00199233	chrX	14979416	25170	C	CC	CCCC
rs6527165	A_20_P00199234	chrX	14996152	65703	C	CC	CCCC
rs12014661	A_20_P00301145	chrX	15100472	27882	A	NN	NN
rs16979775	A_20_P00199241	chrX	15197089	1101	NN	NN	NN
rs6631920	A_20_P00301148	chrX	15219388	19329	A	AA	AAAA
rs6632050	A_20_P00199243	chrX	15247531	119178	G	GG	GGGG
rs6628943	A_20_P00301150	chrX	15308547	124593	G	TG	TTGG
rs2027803	A_20_P00199246	chrX	15407414	68968	NN	CT	CCTT
rs7066548	A_20_P00301154	chrX	15455340	117278	C	CC	CCCC
rs1567894	A_20_P00301155	chrX	15510855	64190	NN	CC	CCCC
rs16979928	A_20_P00301156	chrX	15518515	144774	G	GG	GGGG
rs7060497	A_20_P00199251	chrX	15520542	12826	G	GG	NN
rs4646159	A_20_P00301159	chrX	15595914	115934	C	CC	CCCC
rs4646151	A_20_P00199254	chrX	15598934	110812	NN	NN	NN
rs4830975	A_20_P00301163	chrX	15678182	113623	NN	NN	NN
rs5936013	A_20_P00301164	chrX	15714073	26731	NN	NN	NN
rs1557808	A_20_P00301167	chrX	15829435	53725	G	GG	GGGG
rs5936079	A_20_P00301168	chrX	15957302	149457	T	TT	TTTT
rs4830551	A_20_P00199263	chrX	16000141	104061	G	NN	NN
rs5936088	A_20_P00199264	chrX	16007082	21993	A	NN	NN
rs5936092	A_20_P00301171	chrX	16018163	110987	NN	GG	GGGG
rs2353577	A_20_P00199266	chrX	16167262	120246	G	GG	GGGG
rs7057049	A_20_P00301174	chrX	16178601	10151	G	GG	GGGG
rs2178560	A_20_P00301175	chrX	16195103	78185	A	AA	AAAA
rs1795697	A_20_P00199270	chrX	16244656	115196	NN	CC	CCCC
rs2702239	A_20_P00199271	chrX	16247643	16725	C	CT	CCCC

SNP ID	Probe Name	Chromosome	SNP Position	Feature Number	Father	Mother	Proband
rs6629155	A_20_P00301179	chrX	16592867	51292	G	GG	GGGG
rs6629176	A_20_P00301184	chrX	16790042	173676	NN	TC	TTCC
rs6527727	A_20_P00199279	chrX	16812782	125380	G	NN	NN
rs5924611	A_20_P00199280	chrX	16891997	145061	T	CC	CCCC
rs5969776	A_20_P00199281	chrX	16912703	171827	T	CC	CCCC
rs5969780	A_20_P00199282	chrX	16950941	31721	T	NN	NN
rs16997176	A_20_P00199285	chrX	17149685	171796	T	TT	TTTT
rs3935283	A_20_P00199286	chrX	17194656	69863	T	TT	TTTT
rs7049378	A_20_P00199288	chrX	17272314	150883	C	CC	CCCC
rs5991385	A_20_P00199289	chrX	17292827	134192	NN	CC	CCCC
rs10482252	A_20_P00199290	chrX	17323180	90487	NN	NN	NN
rs5991400	A_20_P00301197	chrX	17367319	79165	NN	CC	CCCC
rs7892218	A_20_P00301199	chrX	17613461	168430	NN	TC	TTCC
rs1007420	A_20_P00199295	chrX	17645693	148352	C	CC	CCCC
rs6633053	A_20_P00199298	chrX	18015809	172761	T	TT	TTTT
rs5955922	A_20_P00301205	chrX	18060091	75276	T	TC	TTCC
rs5955928	A_20_P00199300	chrX	18100807	101860	A	NN	NN
rs2213602	A_20_P00199301	chrX	18108075	54073	A	GG	GGGG
rs7878022	A_20_P00199304	chrX	18229424	114989	T	TT	TTTT
rs7876407	A_20_P00199307	chrX	18592147	101065	C	CC	CCCC
rs7050790	A_20_P00199310	chrX	19007690	69582	C	GC	GGGG
rs6633188	A_20_P00301218	chrX	19066723	162223	NN	CT	CCTT
rs16980971	A_20_P00301219	chrX	19141924	10761	C	NN	NN
rs7058326	A_20_P00301220	chrX	19182475	17887	C	CC	CCCC
rs7877429	A_20_P00199316	chrX	19220745	164254	NN	NN	NN
rs6633217	A_20_P00301224	chrX	19236190	120908	NN	GG	GGGG
rs957026	A_20_P00301225	chrX	19244125	63373	A	CC	CCCC
rs6629328	A_20_P00301226	chrX	19272752	152045	T	TT	TTTT
rs7890403	A_20_P00199321	chrX	19324010	46005	NN	NN	NN
rs1908527	A_20_P00301228	chrX	19327446	1208	G	GG	NN
rs7886893	A_20_P00199324	chrX	19371527	23640	NN	GG	GGGG
rs16981167	A_20_P00301231	chrX	19458472	111884	C	CC	CCCC
rs1458783	A_20_P00301232	chrX	19525525	103204	NN	NN	NN
rs4825304	A_20_P00199327	chrX	19558123	156826	NN	NN	NN
rs7892351	A_20_P00301235	chrX	19770035	11134	NN	NN	NN
rs7885754	A_20_P00301238	chrX	20066256	101312	NN	TG	TTGG
rs7473272	A_20_P00199336	chrX	20419748	42710	NN	AA	AAAA
rs1381265	A_20_P00301243	chrX	20573138	21664	NN	TT	TTTT
rs7056599	A_20_P00199338	chrX	20648599	15564	T	TT	NN
rs17314013	A_20_P00301246	chrX	20694657	14155	T	NN	NN
rs5950285	A_20_P00199343	chrX	20703696	132494	T	TT	TTTT
rs12010979	A_20_P00199344	chrX	20707943	83326	C	CC	CCCC
rs1375088	A_20_P00301252	chrX	20718669	20028	T	TT	TTTT
rs7062942	A_20_P00301253	chrX	20745433	76718	T	TT	NN
rs10521918	A_20_P00301255	chrX	20783199	13347	A	AA	AAAA
rs5990847	A_20_P00199351	chrX	20975464	133496	C	CC	NN
rs1479245	A_20_P00301259	chrX	20999294	38684	A	AA	AAAA

SNP ID	Probe Name	Chromosome	SNP Position	Feature Number	Father	Mother	Proband
rs5951452	A_20_P00199356	chrX	21197058	166861	C	CC	CCCC
rs7058638	A_20_P00199357	chrX	21209663	76376	C	CC	CCCC
rs5951592	A_20_P00199358	chrX	21387110	21258	C	NN	NN
rs5951596	A_20_P00199359	chrX	21489436	20021	NN	GG	GGGG
rs12007745	A_20_P00199360	chrX	21620622	141004	A	AA	AAAA
rs16981614	A_20_P00301267	chrX	21640510	49393	T	TT	TTTT
rs16981641	A_20_P00199363	chrX	21778663	180264	G	GG	GGGG
rs2032426	A_20_P00301270	chrX	21802998	80846	C	NN	NN
rs2032427	A_20_P00301271	chrX	21803107	133965	A	AG	AAGG
rs5951668	A_20_P00199367	chrX	21951065	140671	G	GG	GGGG
rs5951487	A_20_P00301274	chrX	21957802	132501	A	NN	NN
rs12688591	A_20_P00199370	chrX	21983447	105707	G	TT	TTTT
rs7060982	A_20_P00199371	chrX	22080734	139599	T	TT	TTTT
rs7883038	A_20_P00301280	chrX	22178044	157248	C	GC	GGCC
rs7888785	A_20_P00199375	chrX	22223063	136022	C	NN	NN
rs2071584	A_20_P00199376	chrX	22263721	55415	NN	NN	TTTT
rs5951739	A_20_P00199377	chrX	22266890	56953	A	AA	AAAA
rs12689951	A_20_P00199378	chrX	22279058	44405	NN	NN	NN
rs7886569	A_20_P00301285	chrX	22279242	164081	NN	NN	NN
rs12397283	A_20_P00301286	chrX	22285407	170990	A	AA	AAAA
rs16981936	A_20_P00301288	chrX	22302475	128066	T	TT	NN
rs11094824	A_20_P00199383	chrX	22302699	20341	NN	AA	NN
rs4131306	A_20_P00199384	chrX	22324302	66254	NN	NN	CCCC
rs5904524	A_20_P00301291	chrX	22334458	128917	G	NN	NN
rs4279714	A_20_P00301292	chrX	22335497	120850	NN	TG	TTGG
rs6633557	A_20_P00199387	chrX	22383475	102484	C	CC	CCCC
rs4824168	A_20_P00199388	chrX	22384571	53308	NN	NN	NN
rs17249158	A_20_P00301295	chrX	22395968	173104	T	TT	TTTT
rs4828945	A_20_P00301296	chrX	22477903	62816	T	NN	CCCC
rs1395982	A_20_P00301297	chrX	22563242	68615	C	CC	CCCC
rs5925577	A_20_P00199393	chrX	22587683	171213	NN	NN	NN
rs5970612	A_20_P00301301	chrX	22615920	48963	C	CT	CCTT
rs6633604	A_20_P00199396	chrX	22705579	124385	NN	GG	GGGG
rs7882511	A_20_P00199397	chrX	22718634	140550	T	TT	TTTT
rs5926092	A_20_P00199400	chrX	22847005	116438	T	TT	TTTT
rs4828844	A_20_P00301307	chrX	22892898	59584	NN	TT	TTTT
rs12009364	A_20_P00301308	chrX	22898185	165009	T	TT	TTTT
rs6528226	A_20_P00301309	chrX	22961421	46008	C	TT	TTTT
rs5926210	A_20_P00199405	chrX	23036617	132972	C	CC	CCCC
rs12010526	A_20_P00301314	chrX	23078695	35452	A	AA	AAAA
rs12014649	A_20_P00199409	chrX	23100070	955	A	AA	NN
rs5971016	A_20_P00199410	chrX	23113268	110241	T	TC	TTCC
rs16982437	A_20_P00301318	chrX	23128647	28547	G	GG	GGGG
rs6526246	A_20_P00301322	chrX	23230546	19982	NN	CC	CCCC
rs2158450	A_20_P00301325	chrX	23320820	134783	NN	TT	TTTT
rs5971115	A_20_P00199422	chrX	23405681	91456	NN	TG	TTGG
rs12395854	A_20_P00301331	chrX	23436511	141454	NN	TT	TTTT

SNP ID	Probe Name	Chromosome	SNP Position	Feature Number	Father	Mother	Proband
rs6526294	A_20_P00301333	chrX	23498826	110614	C	CG	CCGG
rs7050177	A_20_P00199428	chrX	23525675	64455	NN	NN	NN
rs1033055	A_20_P00199430	chrX	23566531	174016	G	GT	GGTT
rs6526309	A_20_P00199431	chrX	23569512	10473	C	CC	CCCC
rs5970751	A_20_P00301338	chrX	23604315	166967	NN	CC	NN
rs6629698	A_20_P00199434	chrX	23608615	119593	G	AA	AAAA
rs1022101	A_20_P00199435	chrX	23608693	9087	C	TT	TTTT
rs1062104	A_20_P00199438	chrX	23719422	83479	T	TT	TTTT
rs5925593	A_20_P00301345	chrX	23729636	142755	NN	AA	AAAA
rs6526342	A_20_P00199441	chrX	23799738	52220	C	CC	CCCC
rs5970787	A_20_P00301348	chrX	23850793	110582	T	NN	NN
rs5949272	A_20_P00301350	chrX	24080269	2514	NN	CC	CCCC
rs5990070	A_20_P00301352	chrX	24104136	143352	C	CC	CCCC
rs7049514	A_20_P00301353	chrX	24223111	37656	A	AA	AAAA
rs5944762	A_20_P00199451	chrX	24453348	33118	NN	GG	GGGG
rs12013712	A_20_P00199452	chrX	24488963	93055	C	TT	TTTT
rs5986552	A_20_P00301359	chrX	24530942	19484	NN	AG	GGGG
rs7053895	A_20_P00301360	chrX	24538906	81360	G	AA	AAAA
rs1207609	A_20_P00199455	chrX	24558261	109212	G	CC	CCCC
rs1882403	A_20_P00301362	chrX	24580746	147455	NN	NN	NN
rs12846498	A_20_P00301363	chrX	24589130	161069	NN	NN	NN
rs16983194	A_20_P00199458	chrX	24660483	152648	A	AA	AAAA
rs5944665	A_20_P00199459	chrX	24671333	125565	G	AA	AAAA
rs11573344	A_20_P00301367	chrX	24742674	174385	C	NN	CCCC
rs11573349	A_20_P00301368	chrX	24751409	143483	T	TT	TTTT
rs11573388	A_20_P00301369	chrX	24792736	173669	A	AA	AAAA
rs11573404	A_20_P00199464	chrX	24813217	96103	C	NN	NN
rs11573438	A_20_P00199465	chrX	24863702	24791	T	NN	NN
rs16983338	A_20_P00199467	chrX	25174118	108264	A	AA	AAAA
rs9785610	A_20_P00301374	chrX	25197188	27785	T	NN	NN
rs5986750	A_20_P00199469	chrX	25217097	80583	G	NN	NN
rs12014920	A_20_P00199472	chrX	25271625	59263	A	AA	AAAA
rs1481164	A_20_P00301379	chrX	25277477	112665	NN	NN	NN
rs12395722	A_20_P00199475	chrX	25323510	15940	G	GG	GGGG
rs4898227	A_20_P00199476	chrX	25558285	53369	T	GG	GGGG
rs4129664	A_20_P00301384	chrX	25569163	47687	T	TT	TTTT
rs959644	A_20_P00199481	chrX	25805515	174742	C	TC	TTCC
rs5944169	A_20_P00301388	chrX	25814128	82386	NN	GT	GGTT
rs7891638	A_20_P00199483	chrX	25908538	86556	G	GG	GGGG
rs1386634	A_20_P00301390	chrX	25942067	118622	C	CC	CCCC
rs4431758	A_20_P00199487	chrX	26016787	133076	C	CT	CCTT
rs4644429	A_20_P00199488	chrX	26016820	47582	NN	NN	NN
rs5944313	A_20_P00301395	chrX	26148797	81848	G	AA	AAAA
rs4898259	A_20_P00199491	chrX	26158809	43748	NN	NN	CCCC
rs5944341	A_20_P00301399	chrX	26190707	4117	NN	NN	NN
rs4898183	A_20_P00199495	chrX	26485029	60550	NN	CC	CCCC
rs4385610	A_20_P00301402	chrX	26513111	158151	NN	NN	NN

SNP ID	Probe Name	Chromosome	SNP Position	Feature Number	Father	Mother	Proband
rs17348072	A_20_P00199497	chrX	26522421	107616	T	GG	GGGG
rs5944492	A_20_P00199498	chrX	26530440	147450	C	NN	NN
rs12009011	A_20_P00199499	chrX	26549849	93406	NN	NN	NN
rs12389363	A_20_P00199500	chrX	26600620	103942	NN	GG	NN
rs5986332	A_20_P00301407	chrX	26601036	51046	C	TT	TTTT
rs4480265	A_20_P00199502	chrX	26643242	1814	NN	NN	NN
rs12011861	A_20_P00199504	chrX	26754613	27875	A	AA	AAAA
rs5986674	A_20_P00301411	chrX	26829853	21471	NN	GG	GGGG
rs2169974	A_20_P00199506	chrX	26937259	92310	T	NN	NN
rs1479420	A_20_P00199508	chrX	27040824	116451	NN	CC	CCCC
rs5926351	A_20_P00199509	chrX	27043979	81628	C	NN	NN
rs5926392	A_20_P00301417	chrX	27275607	78329	A	AA	AAAA
rs5926779	A_20_P00301419	chrX	27488910	32366	T	NN	NN
rs4829074	A_20_P00301421	chrX	27531964	114551	G	NN	NN
rs12391301	A_20_P00199516	chrX	27573484	143331	C	NN	NN
rs5926843	A_20_P00301425	chrX	27634152	10292	NN	CC	CCCC
rs5971424	A_20_P00301427	chrX	27676641	68285	T	NN	NN
rs2047022	A_20_P00301428	chrX	27693683	141767	T	TT	TTTT
rs1493057	A_20_P00199523	chrX	27710493	98575	NN	AA	AAAA
rs6526711	A_20_P00301430	chrX	27788228	62653	G	GG	NN
rs7888623	A_20_P00199527	chrX	27893877	128177	C	NN	CCCC
rs34563960	A_20_P00199529	chrX	27966366	56163	A	AA	AAAA
rs5971275	A_20_P00199531	chrX	28137324	6341	T	NN	NN
rs10126891	A_20_P00301442	chrX	28490756	134086	T	CC	CCCC
rs7884344	A_20_P00199537	chrX	28504040	119220	G	GG	GGGG
rs7054195	A_20_P00199538	chrX	28550863	89524	C	NN	CCCC
rs7056889	A_20_P00199541	chrX	28634463	126148	C	CC	CCCC
rs5985883	A_20_P00199542	chrX	28639740	163811	NN	NN	NN
rs10521946	A_20_P00301449	chrX	28770685	27876	G	NN	NN
rs5943547	A_20_P00199544	chrX	28794053	11351	NN	AG	AAGG
rs4893556	A_20_P00199545	chrX	28872232	39704	G	NN	GGGG
rs1318832	A_20_P00199546	chrX	28873495	88453	C	TT	TTTT
rs12390283	A_20_P00301453	chrX	28889826	176093	C	NN	NN
rs1392713	A_20_P00199548	chrX	29021738	95894	NN	NN	NN
rs4353014	A_20_P00301456	chrX	29182301	168520	T	TT	TTTT
rs12014119	A_20_P00199551	chrX	29333886	64247	C	CT	CCTT
rs6526848	A_20_P00301458	chrX	29344356	169615	C	TC	TTCC
rs5927700	A_20_P00199553	chrX	29378683	68700	T	NN	NN
rs5971905	A_20_P00199554	chrX	29415656	82853	G	GG	GGGG
rs6628429	A_20_P00301461	chrX	29465999	143238	G	GG	GGGG
rs12008170	A_20_P00301464	chrX	29566533	30357	NN	AA	AAAA
rs7051233	A_20_P00199559	chrX	29647078	36042	G	GG	GGGG
rs7879913	A_20_P00199560	chrX	29700416	96021	T	CT	CCTT
rs4829265	A_20_P00199561	chrX	29704536	129165	NN	AG	AAGG
rs6628466	A_20_P00301468	chrX	29707179	99455	A	NN	NN
rs6526916	A_20_P00301469	chrX	29708619	146852	G	NN	NN
rs7876313	A_20_P00301471	chrX	29768119	15349	NN	TT	TTTT

SNP ID	Probe Name	Chromosome	SNP Position	Feature Number	Father	Mother	Proband
rs11095160	A_20_P00301472	chrX	29792108	121301	A	AG	AAGG
rs16998033	A_20_P00301474	chrX	29873794	113028	T	TT	TTTT
rs7891129	A_20_P00199569	chrX	29884207	163156	NN	NN	NN
rs2529550	A_20_P00301477	chrX	30250326	17388	G	AG	AAGG
rs7891921	A_20_P00301484	chrX	30486021	162638	C	CC	CCCC
rs6526983	A_20_P00301485	chrX	30574075	32574	A	AA	AAAA
rs758625	A_20_P00301486	chrX	30579655	14072	A	GG	GGGG
rs5927575	A_20_P00199581	chrX	30643333	53717	G	NN	NN
rs17329748	A_20_P00301490	chrX	30762377	68560	C	CC	CCCC
rs5926963	A_20_P00301491	chrX	30827846	74184	NN	NN	NN
rs6631213	A_20_P00301492	chrX	30970973	8366	A	AA	AAAA
rs5927643	A_20_P00301493	chrX	30978304	154899	NN	TT	TTTT
rs5972323	A_20_P00301494	chrX	31184563	54201	G	CG	CCGG
rs16989410	A_20_P00199589	chrX	31186307	166999	A	AA	AAAA
rs1921395	A_20_P00301496	chrX	31195351	56879	G	CG	CCGG
rs5972366	A_20_P00301498	chrX	31324175	96299	C	CC	CCCC
rs12008257	A_20_P00199595	chrX	31336105	83579	NN	CC	CCCC
rs16998177	A_20_P00199596	chrX	31336410	30644	A	AA	AAAA
rs2692976	A_20_P00199597	chrX	31355988	170314	NN	AA	AAAA
rs6628597	A_20_P00199598	chrX	31382037	73106	NN	TC	TTCC
rs5972381	A_20_P00301505	chrX	31407068	119610	T	TT	TTTT
rs17340791	A_20_P00301507	chrX	31477547	171942	NN	NN	NN
rs12687514	A_20_P00199602	chrX	31521411	170664	NN	NN	CCCC
rs4829105	A_20_P00301509	chrX	31604807	164583	NN	NN	TTTT
rs716354	A_20_P00199604	chrX	31634092	141422	NN	NN	NN
rs5971585	A_20_P00301511	chrX	31662036	146906	G	GG	GGGG
rs16989767	A_20_P00199606	chrX	31674988	33985	A	AA	AAAA
rs5972435	A_20_P00301513	chrX	31730752	40119	T	TT	TTTT
rs5927819	A_20_P00199608	chrX	31745485	170385	NN	NN	NN
rs17340949	A_20_P00301515	chrX	31795512	30592	NN	NN	NN
rs16998232	A_20_P00301517	chrX	31912609	157480	C	CC	CCCC
rs7879040	A_20_P00301519	chrX	31933609	88763	G	GG	GGGG
rs870133	A_20_P00301520	chrX	31951698	37428	G	NN	NN
rs5972470	A_20_P00199616	chrX	32009476	125235	G	AA	AAAA
rs6628669	A_20_P00301524	chrX	32037792	53791	A	AA	NN
rs1293879	A_20_P00199619	chrX	32039589	140517	NN	TT	TTTT
rs1921375	A_20_P00199621	chrX	32057482	131985	C	CC	CCCC
rs5972488	A_20_P00199622	chrX	32102807	8352	NN	TT	TTTT
rs6631486	A_20_P00301529	chrX	32102976	95539	NN	TT	TTTT
rs7882019	A_20_P00199624	chrX	32116068	77994	C	TT	TTTT
rs2685907	A_20_P00199625	chrX	32194441	168393	NN	CC	CCCC
rs1379109	A_20_P00199627	chrX	32212041	72437	C	CC	CCCC
rs1325574	A_20_P00301535	chrX	32266542	160256	NN	AG	AAGG
rs331338	A_20_P00199630	chrX	32298707	57507	NN	CC	CCCC
rs7887340	A_20_P00199631	chrX	32308742	129571	C	CC	CCCC
rs331332	A_20_P00199632	chrX	32311601	56134	A	AG	AAGG
rs2038354	A_20_P00301539	chrX	32393558	44646	NN	NN	NN

SNP ID	Probe Name	Chromosome	SNP Position	Feature Number	Father	Mother	Proband
rs5972549	A_20_P00199634	chrX	32400177	58971	C	GC	GGCC
rs5971631	A_20_P00199635	chrX	32413107	175334	NN	NN	NN
rs10126490	A_20_P00301542	chrX	32561531	91727	NN	NN	NN
rs813974	A_20_P00301544	chrX	32577452	106430	NN	CG	CCGG
rs12557761	A_20_P00301547	chrX	32642257	180350	C	CC	CCCC
rs5928067	A_20_P00301549	chrX	32693029	35403	NN	GG	GGGG
rs6631620	A_20_P00199644	chrX	32705790	20701	A	NN	NN
rs5972664	A_20_P00301551	chrX	32777635	14236	A	AA	AAAA
rs2855693	A_20_P00301552	chrX	32780583	161000	C	NN	CCCC
rs1304485	A_20_P00301554	chrX	32809113	106031	NN	TC	TTCC
rs5971665	A_20_P00301555	chrX	32866065	69791	A	GA	GGAA
rs5928111	A_20_P00199650	chrX	32931528	96522	NN	AA	AAAA
rs2145207	A_20_P00301557	chrX	32966821	142034	NN	NN	NN
rs5928127	A_20_P00199652	chrX	32994802	103506	NN	NN	NN
rs6631710	A_20_P00199654	chrX	33024163	86216	G	GG	AAGG
rs12849156	A_20_P00199655	chrX	33024720	77623	T	TT	TTTT
rs5928153	A_20_P00301562	chrX	33040341	149162	G	GG	NN
rs2025664	A_20_P00301565	chrX	33056308	149305	A	AG	AAGG
rs5972776	A_20_P00301568	chrX	33219599	18745	A	AA	AAAA
rs971642	A_20_P00301569	chrX	33224520	79761	G	NN	NN
rs16990960	A_20_P00301570	chrX	33249313	44369	T	NN	NN
rs5928280	A_20_P00301573	chrX	33486083	83873	C	CC	NN
rs5928283	A_20_P00199668	chrX	33501853	143268	C	CC	CCCC
rs5928305	A_20_P00199669	chrX	33521250	139846	G	GT	GGTT
rs1464244	A_20_P00199673	chrX	33657239	97272	T	NN	TTTT
rs12007168	A_20_P00199674	chrX	33716777	112332	T	TT	TTTT
rs5928357	A_20_P00199675	chrX	33774480	92721	NN	AG	AAGG
rs3006150	A_20_P00199679	chrX	34021156	117123	G	AG	AAGG
rs4829324	A_20_P00301592	chrX	34216535	74865	C	NN	NN
rs5928473	A_20_P00199687	chrX	34235392	117701	NN	CC	CCCC
rs5973132	A_20_P00301594	chrX	34255430	24094	T	AA	AAAA
rs16991320	A_20_P00301596	chrX	34344041	102211	A	AA	AAAA
rs12010142	A_20_P00199691	chrX	34355538	87856	C	CC	CCCC
rs5927267	A_20_P00301598	chrX	34383989	128315	NN	CC	CCCC
rs4335292	A_20_P00301599	chrX	34399257	120762	T	AA	AAAA
rs5973200	A_20_P00301600	chrX	34482953	33826	A	NN	NN
rs5971871	A_20_P00199697	chrX	34517386	166437	C	CC	CCCC
rs6628869	A_20_P00301604	chrX	34566759	134472	NN	NN	NN
rs4408051	A_20_P00301605	chrX	34672709	61724	NN	NN	NN
rs16991505	A_20_P00199702	chrX	34762403	94007	G	GG	GGGG
rs17244267	A_20_P00301610	chrX	34919806	38661	T	TT	TTTT
rs3128068	A_20_P00301611	chrX	34944998	115312	C	CC	CCCC
rs3109514	A_20_P00199706	chrX	34992270	66438	G	GG	GGGG
rs4460544	A_20_P00301613	chrX	35003994	111923	C	CC	CCCC
rs4468066	A_20_P00199708	chrX	35018159	112972	NN	AA	AAAA
rs3124845	A_20_P00199709	chrX	35025830	2909	A	AA	AAAA
rs11796025	A_20_P00199712	chrX	35203303	19079	NN	CC	CCCC

SNP ID	Probe Name	Chromosome	SNP Position	Feature Number	Father	Mother	Proband
rs5928767	A_20_P00199714	chrX	35252589	151505	NN	TT	TTTT
rs5927392	A_20_P00301622	chrX	35357940	130718	G	AA	AAAA
rs1037970	A_20_P00301623	chrX	35370744	157506	T	AA	AAAA
rs1462703	A_20_P00301624	chrX	35380779	67764	C	TT	TTTT
rs5927399	A_20_P00301625	chrX	35434714	85412	C	TT	TTTT
rs5927404	A_20_P00199720	chrX	35462703	49380	NN	NN	AAAA
rs5973375	A_20_P00301627	chrX	35489061	585	A	CC	CCCC
rs5928966	A_20_P00199724	chrX	35692917	18445	C	NN	NN
rs5971974	A_20_P00199725	chrX	35812525	80296	G	GG	GGGG
rs16992135	A_20_P00301633	chrX	35819858	43323	C	CC	CCCC
rs1571478	A_20_P00199729	chrX	35824841	77093	G	NN	NN
rs2878684	A_20_P00199731	chrX	35886470	129048	G	NN	NN
rs5973517	A_20_P00199732	chrX	35888009	49098	G	NN	NN
rs5972010	A_20_P00199735	chrX	36018434	105484	G	GG	NN
rs11095425	A_20_P00199736	chrX	36133458	113678	C	CC	CCCC
rs4132845	A_20_P00199738	chrX	36286468	48531	A	AA	AAAA
rs16987455	A_20_P00199739	chrX	36315412	108252	NN	NN	CCCC
rs5973644	A_20_P00301646	chrX	36341688	43031	T	TT	TTTT
rs16987499	A_20_P00199741	chrX	36463239	167697	A	AA	AAAA
rs16987510	A_20_P00199742	chrX	36500702	180118	A	AA	AAAA
rs7049360	A_20_P00199743	chrX	36516555	24573	NN	NN	AAAA
rs16987549	A_20_P00301650	chrX	36577165	17076	A	AA	AAAA
rs6527592	A_20_P00301652	chrX	36623493	141849	T	TT	TTTT
rs12861803	A_20_P00301653	chrX	36625588	165405	C	CC	CCCC
rs12839347	A_20_P00301654	chrX	36626863	127472	A	AA	AAAA
rs4829412	A_20_P00301655	chrX	36639717	177119	C	CT	CCCC
rs12006776	A_20_P00301658	chrX	36691117	32028	T	TT	TTTT
rs4595279	A_20_P00199753	chrX	36717825	75299	A	AA	AAAA
rs28683748	A_20_P00199755	chrX	36792374	76989	T	TT	TTTT
rs17145356	A_20_P00301663	chrX	37167612	166264	NN	NN	NN
rs5963672	A_20_P00301666	chrX	37393218	98508	G	GG	GGGG
rs3117499	A_20_P00301667	chrX	37482090	161836	T	NN	TTTT
rs3117506	A_20_P00199762	chrX	37496283	96320	C	CC	CCCC
rs6520785	A_20_P00301669	chrX	37644425	85254	C	NN	NN
rs5963309	A_20_P00199764	chrX	37659056	23689	A	AA	AAAA
rs12011274	A_20_P00301671	chrX	37664978	100132	A	AA	AAAA
rs5963327	A_20_P00301673	chrX	37673219	134726	G	GG	GGGG
rs7881026	A_20_P00199769	chrX	37700954	173509	NN	NN	NN
rs11771	A_20_P00301676	chrX	37701048	94803	G	AA	AAAA
rs7051301	A_20_P00199771	chrX	37762072	100251	T	TT	TTTT
rs7882520	A_20_P00301680	chrX	37858476	48382	G	GG	GGGG
rs1884691	A_20_P00199775	chrX	37864749	41829	NN	NN	TTTT
rs1003751	A_20_P00199776	chrX	37911800	26417	G	GG	NN
rs7878366	A_20_P00301683	chrX	37926012	166906	NN	TT	TTTT
rs4827339	A_20_P00199779	chrX	38063433	151233	NN	GC	GGCC
rs2224559	A_20_P00199780	chrX	38067679	44337	C	CC	CCCC
rs5918507	A_20_P00199781	chrX	38088814	163667	T	TT	NN

SNP ID	Probe Name	Chromosome	SNP Position	Feature Number	Father	Mother	Proband
rs10284165	A_20_P00199782	chrX	38107183	119892	NN	NN	GGGG
rs11795845	A_20_P00199783	chrX	38140842	8788	A	NN	NN
rs5963398	A_20_P00301690	chrX	38171761	27003	C	TT	TTTT
rs5963408	A_20_P00301692	chrX	38210814	43792	G	AA	AAAA
rs17144919	A_20_P00301693	chrX	38225648	104247	NN	TT	TTTT
rs5963440	A_20_P00301694	chrX	38298009	157806	T	TC	TTCC
rs5963054	A_20_P00301695	chrX	38386447	77072	T	NN	NN
rs5963481	A_20_P00199790	chrX	38390656	55817	G	AA	AAAA
rs6520473	A_20_P00301698	chrX	38397317	150491	A	GG	GGGG
rs5917213	A_20_P00301700	chrX	38483180	116411	G	GG	GGGG
rs7049900	A_20_P00199795	chrX	38526537	52790	C	CC	CCCC
rs5917617	A_20_P00199796	chrX	38534664	28259	NN	AG	AAGG
rs17145123	A_20_P00199797	chrX	38583246	176447	T	TT	TTTT
rs198786	A_20_P00301704	chrX	38673079	161615	NN	CC	CCCC
rs199867	A_20_P00301705	chrX	38737343	155766	NN	AG	AAGG
rs1546843	A_20_P00301706	chrX	38843505	29480	NN	NN	NN
rs5963615	A_20_P00301708	chrX	39096656	146629	T	TT	TTTT
rs2221303	A_20_P00199803	chrX	39180956	1495	C	NN	NN
rs12837604	A_20_P00301713	chrX	39253392	17708	NN	NN	NN
rs1472041	A_20_P00199808	chrX	39292699	86235	A	AA	AAAA
rs1318833	A_20_P00301715	chrX	39345375	61165	NN	CC	CCCC
rs6520585	A_20_P00301717	chrX	39391447	60739	NN	NN	NN
rs207045	A_20_P00199814	chrX	39553429	169126	C	NN	NN
rs6609050	A_20_P00301724	chrX	39915719	48471	A	AG	AAGG
rs4072310	A_20_P00199828	chrX	40505730	51896	C	CC	CCCC
rs6520674	A_20_P00199829	chrX	40537738	81498	C	CC	CCCC
rs6609087	A_20_P00199830	chrX	40565844	90870	C	NN	NN
rs10127045	A_20_P00199831	chrX	40745226	88751	A	AA	AAAA
rs34025258	A_20_P00199832	chrX	40773347	105351	NN	NN	NN
rs6417860	A_20_P00199834	chrX	40892018	161376	A	GG	GGGG
rs17145980	A_20_P00199838	chrX	41073148	70678	A	NN	AAAA
rs5918140	A_20_P00301746	chrX	41117531	164179	NN	NN	NN
rs1467317	A_20_P00199842	chrX	41173758	108670	NN	NN	GGGG
rs787088	A_20_P00199843	chrX	41235894	53385	NN	NN	NN
rs787086	A_20_P00199844	chrX	41238242	86601	G	AG	AAGG
rs5964012	A_20_P00301751	chrX	41421540	135004	NN	GG	GGGG
rs6651765	A_20_P00301754	chrX	41498802	30971	A	AA	AAAA
rs12396277	A_20_P00301755	chrX	41568451	23019	C	NN	NN
rs7059771	A_20_P00301756	chrX	41840784	89433	T	TT	NN
rs6609184	A_20_P00199852	chrX	41918167	121320	C	TT	TTTT
rs1074596	A_20_P00199853	chrX	41945535	125071	NN	CC	CCCC
rs5918283	A_20_P00199854	chrX	41946526	107030	T	TT	TTTT
rs5918309	A_20_P00199857	chrX	42095467	29124	NN	CC	CCCC
rs5918312	A_20_P00199858	chrX	42105345	166697	NN	CC	CCCC
rs5963335	A_20_P00199860	chrX	42347020	37365	C	CC	CCCC
rs5963348	A_20_P00301767	chrX	42614602	160577	C	GC	GGCC
rs7884120	A_20_P00301768	chrX	42615589	16819	G	AG	AAGG

SNP ID	Probe Name	Chromosome	SNP Position	Feature Number	Father	Mother	Proband
rs2157385	A_20_P00199864	chrX	42659478	144117	C	CA	CCAA
rs5991588	A_20_P00199865	chrX	42662929	27347	C	NN	NN
rs12012123	A_20_P00301773	chrX	42724042	78024	G	GG	GGGG
rs5991696	A_20_P00301774	chrX	42743203	66392	C	CC	CCCC
rs7065315	A_20_P00199871	chrX	42826643	18921	T	TT	TTTT
rs6520824	A_20_P00199872	chrX	42835110	85061	NN	CG	CCGG
rs7882439	A_20_P00301779	chrX	42836892	167628	G	NN	NN
rs5950942	A_20_P00301780	chrX	42927865	26470	A	CA	CCAA
rs5991593	A_20_P00199876	chrX	42951395	135191	A	AA	AAAA
rs5950955	A_20_P00199877	chrX	42979747	121973	G	NN	NN
rs1155699	A_20_P00199880	chrX	43250720	88127	A	AG	AAGG
rs1339774	A_20_P00199883	chrX	43359055	53384	NN	AC	AACC
rs1339777	A_20_P00301791	chrX	43437026	151638	A	NN	NN
rs4986551	A_20_P00199887	chrX	43447376	16976	G	NN	NN
rs5905702	A_20_P00199888	chrX	43518188	156171	NN	NN	NN
rs7882699	A_20_P00301795	chrX	43535927	167997	NN	NN	NN
rs3027403	A_20_P00199892	chrX	43595678	79958	C	CC	CCCC
rs3027431	A_20_P00199893	chrX	43623084	49517	NN	NN	GGGG
rs5952696	A_20_P00199894	chrX	43639615	173375	T	TT	TTTT
rs3027454	A_20_P00199895	chrX	43658375	26537	T	NN	NN
rs5905703	A_20_P00199897	chrX	43993347	31365	T	CT	CCTT
rs5906920	A_20_P00199899	chrX	44191892	26304	T	CT	CCTT
rs7054290	A_20_P00199901	chrX	44216570	127144	T	TT	TTTT
rs12389196	A_20_P00199904	chrX	44396401	172332	T	NN	NN
rs7890692	A_20_P00301811	chrX	44408030	15744	A	AA	AAAA
rs4824519	A_20_P00301814	chrX	44650253	105358	NN	NN	NN
rs6651830	A_20_P00301816	chrX	44917568	23934	A	AA	AAAA
rs5905427	A_20_P00301817	chrX	45047609	19225	C	CC	CCCC
rs7051979	A_20_P00301820	chrX	45212306	55989	T	TT	TTTT
rs7879677	A_20_P00301821	chrX	45243640	164104	C	CC	NN
rs5906003	A_20_P00301822	chrX	45254633	88235	C	NN	NN
rs5952326	A_20_P00301825	chrX	45558094	108722	NN	NN	NN
rs2223509	A_20_P00301826	chrX	45657565	147541	G	GG	GGGG
rs4824560	A_20_P00199921	chrX	45657819	18419	NN	TT	TTTT
rs5906125	A_20_P00199922	chrX	45813458	84614	G	NN	NN
rs7878147	A_20_P00199925	chrX	46159107	147422	G	GG	GGGG
rs6611237	A_20_P00199930	chrX	46323202	87268	NN	AA	AAAA
rs5952897	A_20_P00301837	chrX	46382502	150049	C	CC	CCCC
rs12014644	A_20_P00199932	chrX	46460615	62134	C	CC	CCCC
rs5906259	A_20_P00301839	chrX	46561566	162205	A	NN	NN
rs6521151	A_20_P00199934	chrX	46636971	104315	C	CC	CCCC
rs17147434	A_20_P00199935	chrX	46791335	27554	A	AA	AAAA
rs12007789	A_20_P00301843	chrX	47067554	67532	T	TT	TTTT
rs7050878	A_20_P00199938	chrX	47242121	72441	T	CC	CCCC
rs3810664	A_20_P00199939	chrX	47306422	10428	T	CC	CCCC
rs6520267	A_20_P00301846	chrX	47313909	154218	NN	NN	NN
rs6608724	A_20_P00301847	chrX	47323527	148404	A	GG	GGGG

SNP ID	Probe Name	Chromosome	SNP Position	Feature Number	Father	Mother	Proband
rs1207469	A_20_P00301851	chrX	47673984	116018	G	GG	GGGG
rs1207486	A_20_P00301853	chrX	47692606	179070	NN	CC	CCCC
rs5906515	A_20_P00199951	chrX	47759571	159368	NN	NN	NN
rs566171	A_20_P00301862	chrX	47840399	60958	NN	NN	NN
rs17148218	A_20_P00301865	chrX	47980874	152008	NN	NN	NN
rs6608782	A_20_P00301866	chrX	48084340	50792	NN	AA	AAAA
rs11091235	A_20_P00301869	chrX	48364635	125394	G	NN	NN
rs11795513	A_20_P00199964	chrX	48395882	20069	T	NN	NN
rs235838	A_20_P00199966	chrX	48464413	140549	G	NN	NN
rs7878739	A_20_P00301873	chrX	48467186	106262	C	CC	CCCC
rs2238978	A_20_P00199968	chrX	48769623	39524	NN	NN	NN
rs2283733	A_20_P00301877	chrX	49073385	119595	NN	AG	AAGG
rs2091979	A_20_P00301880	chrX	49398637	151123	T	GG	GGGG
rs4824807	A_20_P00301882	chrX	49504886	88551	T	TT	TTTT
rs6608946	A_20_P00199979	chrX	49634445	146318	G	NN	GGGG
rs10521459	A_20_P00199980	chrX	49684040	7390	G	NN	NN
rs17174048	A_20_P00199981	chrX	49747856	150455	NN	AA	AAAA
rs12014289	A_20_P00199982	chrX	49767033	133799	A	NN	AAAA
rs7881959	A_20_P00199984	chrX	49912886	48439	G	GG	GGGG
rs7885537	A_20_P00301894	chrX	50219446	64275	C	CC	CCCC
rs4826635	A_20_P00199989	chrX	50222099	9208	C	CC	CCCC
rs5915274	A_20_P00199990	chrX	50325888	17589	C	CC	CCCC
rs2295544	A_20_P00199991	chrX	50341598	52509	A	AA	AAAA
rs5915278	A_20_P00199992	chrX	50343569	84745	A	NN	NN
rs7880497	A_20_P00301899	chrX	50357704	170396	G	GG	GGGG
rs7879616	A_20_P00199994	chrX	50458094	62779	G	GG	GGGG
rs2382657	A_20_P00199996	chrX	50516715	73033	T	TT	TTTT
rs2382675	A_20_P00301903	chrX	50578528	33263	G	GG	GGGG
rs17003223	A_20_P00301904	chrX	50673472	54569	NN	NN	NN
rs7889082	A_20_P00301905	chrX	50728694	174998	A	AA	AAAA
rs5915383	A_20_P00301906	chrX	50768860	89334	NN	NN	NN
rs12390030	A_20_P00301910	chrX	50892415	159466	T	NN	NN
rs11091750	A_20_P00301915	chrX	51380072	2491	G	GG	GGGG
rs11796701	A_20_P00301916	chrX	51523334	114570	A	AA	AAAA
rs5991758	A_20_P00200012	chrX	51694778	41569	T	TT	TTTT
rs10217920	A_20_P00301920	chrX	51850190	41233	T	TT	TTTT
rs7473361	A_20_P00200015	chrX	52093183	14281	C	CC	NN
rs2148098	A_20_P00200017	chrX	53150837	2918	T	NN	TTTT
rs1536249	A_20_P00301924	chrX	53284641	22376	NN	NN	TTTT
rs2497955	A_20_P00200019	chrX	53346686	87893	C	CC	CCCC
rs1018657	A_20_P00301926	chrX	53384103	76831	NN	NN	NN
rs12380898	A_20_P00301928	chrX	53789479	156841	T	CT	CCTT
rs7061449	A_20_P00301931	chrX	54019295	135552	C	CC	CCCC
rs17002479	A_20_P00200026	chrX	54064786	65465	C	CC	CCCC
rs2516036	A_20_P00200027	chrX	54198702	53521	T	TT	TTTT
rs17002582	A_20_P00200030	chrX	54756814	40547	A	AA	AAAA
rs12007444	A_20_P00301939	chrX	55010947	168281	T	TT	TTTT

SNP ID	Probe Name	Chromosome	SNP Position	Feature Number	Father	Mother	Proband
rs12558597	A_20_P00301942	chrX	55391736	106393	T	CT	CCTT
rs5914342	A_20_P00200038	chrX	55433576	2646	G	GG	GGGG
rs5914366	A_20_P00200039	chrX	55467907	129224	A	AC	AACC
rs5914428	A_20_P00200042	chrX	55746426	119579	A	AC	AACC
rs5960542	A_20_P00200045	chrX	55981858	174491	NN	NN	NN
rs2375580	A_20_P00200046	chrX	55988801	102000	A	AA	AAAA
rs5960578	A_20_P00200049	chrX	56058835	175985	A	AA	AAAA
rs5960146	A_20_P00301956	chrX	56111278	58827	A	AA	AAAA
rs5960644	A_20_P00200051	chrX	56169620	139954	NN	NN	NN
rs5914644	A_20_P00200053	chrX	56427996	167826	C	AC	AACC
rs5914776	A_20_P00200056	chrX	56753179	32836	G	TG	TTGG
rs1974070	A_20_P00301964	chrX	56811462	82290	C	TC	TTCC
rs5960809	A_20_P00200059	chrX	56838991	107320	C	CT	CCTT
rs5914943	A_20_P00301968	chrX	57287302	45143	G	GA	GGAA
rs5914091	A_20_P00200063	chrX	57394102	67489	NN	NN	NN
rs1116848	A_20_P00200065	chrX	57481477	92868	C	CC	CCCC
rs2982254	A_20_P00301972	chrX	57615208	148402	NN	NN	NN
rs7049547	A_20_P00301977	chrX	57999932	106808	G	GG	GGGG
rs7891447	A_20_P00200074	chrX	62850896	74784	C	NN	CCCC
rs7881194	A_20_P00301982	chrX	63663978	159331	A	AA	AAAA
rs16991207	A_20_P00301984	chrX	63791143	86187	T	TT	TTTT
rs7057565	A_20_P00301985	chrX	63812456	110055	G	GG	NN
rs6624737	A_20_P00200081	chrX	64076985	1469	NN	GG	GGGG
rs7886111	A_20_P00301989	chrX	64579227	56614	G	GG	GGGG
rs6624812	A_20_P00301992	chrX	64938037	123884	T	TT	TTTT
rs5964462	A_20_P00301995	chrX	65083346	175353	C	CC	CCCC
rs12387084	A_20_P00301996	chrX	65089788	132258	G	GG	GGGG
rs5965047	A_20_P00200091	chrX	65124301	118785	C	CC	CCCC
rs6525038	A_20_P00301999	chrX	65193018	87520	A	AA	AAAA
rs7879464	A_20_P00302000	chrX	65230414	87827	C	CC	CCCC
rs4827470	A_20_P00200098	chrX	65627021	162216	T	NN	NN
rs5965243	A_20_P00200099	chrX	65753462	24489	A	AA	AAAA
rs11093964	A_20_P00302008	chrX	66018207	142645	A	AA	AAAA
rs1334763	A_20_P00200103	chrX	66085317	107269	T	TT	TTTT
rs7049401	A_20_P00200104	chrX	66090479	123604	C	NN	NN
rs5965331	A_20_P00302011	chrX	66109492	33479	C	CC	CCCC
rs7440	A_20_P00200108	chrX	66949722	701	T	NN	NN
rs7064585	A_20_P00302015	chrX	66956221	157778	T	TT	TTTT
rs2885908	A_20_P00200111	chrX	67146381	67283	NN	TT	NN
rs5918799	A_20_P00200114	chrX	67199956	21967	NN	AA	NN
rs913310	A_20_P00200117	chrX	67370962	68016	C	CC	CCCC
rs6625312	A_20_P00302026	chrX	67625009	29573	A	AA	AAAA
rs5919559	A_20_P00302027	chrX	67645007	70537	A	AA	AAAA
rs5937126	A_20_P00200126	chrX	68187621	144411	NN	GG	CCCC
rs2136826	A_20_P00302036	chrX	¥68,223,133	¥27,499	C	CC	TTTT
rs5937151	A_20_P00302037	chrX	68334776	15374	NN	CC	CCCC
rs7055857	A_20_P00200132	chrX	68352433	21648	G	GG	GGGG

SNP ID	Probe Name	Chromosome	SNP Position	Feature Number	Father	Mother	Proband
rs7062312	A_20_P00302040	chrX	68447052	155002	G	GG	AAAA
rs5937186	A_20_P00200135	chrX	68460486	3423	G	GG	GGGG
rs7064760	A_20_P00200137	chrX	68472583	28742	NN	NN	TTTT
rs16990918	A_20_P00302045	chrX	68569057	152784	C	NN	NN
rs12844850	A_20_P00200141	chrX	68754737	169144	NN	NN	NN
rs6525311	A_20_P00302048	chrX	68761102	53476	NN	NN	NN
rs5980827	A_20_P00302050	chrX	68831012	32981	NN	NN	NN
rs12156697	A_20_P00302053	chrX	69049912	21929	G	GA	AAAA
rs5936781	A_20_P00200148	chrX	69082958	121722	C	NN	NN
rs5980674	A_20_P00200150	chrX	69190475	43045	A	NN	NN
rs5980896	A_20_P00302060	chrX	69299947	108372	NN	CC	CCCC
rs1152182	A_20_P00302061	chrX	69423721	31904	T	TT	TTCC
rs1152198	A_20_P00200156	chrX	69456432	54690	NN	AA	AAAA
rs3818862	A_20_P00200157	chrX	69489530	93899	NN	NN	NN
rs5936879	A_20_P00200160	chrX	69579377	81079	NN	AT	AAAA
rs6525367	A_20_P00200161	chrX	69690459	4280	G	AG	AAGG
rs4844242	A_20_P00302070	chrX	69815365	162858	NN	TC	TTCC
rs16991279	A_20_P00200166	chrX	70023114	102743	A	AA	AAGG
rs17216855	A_20_P00200167	chrX	70092876	40921	A	NN	AAAA
rs5937047	A_20_P00302076	chrX	70202127	24895	NN	NN	NN
rs5981072	A_20_P00200172	chrX	70319542	39100	NN	CC	CCCC
rs5981109	A_20_P00200173	chrX	70606208	92137	G	GG	GGGG
rs12387850	A_20_P00200174	chrX	71024480	83938	A	AA	AAAA
rs6418441	A_20_P00200176	chrX	71094742	75103	G	GG	GGGG
rs5951180	A_20_P00302083	chrX	71163204	52201	NN	GG	GGGG
rs7474004	A_20_P00302085	chrX	71193929	154478	G	GG	GGGG
rs4986637	A_20_P00302086	chrX	71205538	43547	T	TT	TTTT
rs5951249	A_20_P00302087	chrX	71233094	40899	T	NN	TTTT
rs3761521	A_20_P00302090	chrX	71499347	68335	NN	NN	NN
rs12007555	A_20_P00200188	chrX	71651181	17235	C	CC	GGGG
rs12009360	A_20_P00200189	chrX	71730990	96127	NN	NN	GGGG
rs7054230	A_20_P00200190	chrX	71819691	49226	G	GG	GGGG
rs7056520	A_20_P00200192	chrX	71840354	106476	T	TT	CCCC
rs5981773	A_20_P00302099	chrX	72313420	67177	G	GG	AAAA
rs4892401	A_20_P00200195	chrX	72335915	23263	C	NN	NN
rs2362989	A_20_P00200197	chrX	72375918	163963	C	CC	CCCC
rs2075528	A_20_P00302106	chrX	72674054	16662	NN	NN	NN
rs798623	A_20_P00200201	chrX	73003744	137929	A	AA	GGGG
rs1009948	A_20_P00302108	chrX	73053673	13159	C	CC	CCCC
rs12688161	A_20_P00302109	chrX	73304834	175768	NN	NN	NN
rs174185	A_20_P00302111	chrX	73473028	47791	A	AA	AAAA
rs1263251	A_20_P00302112	chrX	73558940	36943	NN	CC	CCCC
rs5981652	A_20_P00302113	chrX	73809926	52296	NN	NN	NN
rs5937293	A_20_P00302114	chrX	73927731	101314	A	AA	AAAA
rs6647577	A_20_P00200210	chrX	74132540	44111	NN	NN	NN
rs5981755	A_20_P00200211	chrX	74267742	92831	NN	NN	NN
rs6647650	A_20_P00200214	chrX	74387243	171902	NN	NN	NN

SNP ID	Probe Name	Chromosome	SNP Position	Feature Number	Father	Mother	Proband
rs5981818	A_20_P00302121	chrX	74444361	48199	T	TT	TTTT
rs7885952	A_20_P00302122	chrX	74506413	64879	T	TT	TTTT
rs12861282	A_20_P00302123	chrX	74602803	66375	T	TT	TTTT
rs5981378	A_20_P00302126	chrX	74827971	3758	C	CC	CCCC
rs4892411	A_20_P00200221	chrX	74843150	39198	A	AA	GGGG
rs12009122	A_20_P00302129	chrX	74844859	71156	G	NN	NN
rs5981380	A_20_P00200225	chrX	74875138	43049	T	TT	CCCC
rs4301570	A_20_P00302137	chrX	75221873	173980	C	CC	CCCC
rs4892427	A_20_P00302139	chrX	75358204	174677	NN	TT	TTTT
rs12164341	A_20_P00200236	chrX	75403652	145045	NN	NN	NN
rs5982141	A_20_P00200238	chrX	75641695	85549	NN	NN	NN
rs6648130	A_20_P00302147	chrX	75906726	87504	NN	NN	NN
rs234252	A_20_P00302148	chrX	75956193	163033	G	GG	CCCC
rs5937619	A_20_P00200246	chrX	76069743	79177	NN	GG	GGGG
rs5938788	A_20_P00200249	chrX	76198339	64611	T	TT	TTTT
rs5938835	A_20_P00200250	chrX	76286322	63875	NN	NN	TTTT
rs3131482	A_20_P00302157	chrX	76324236	132939	NN	GG	GGGG
rs1597260	A_20_P00200252	chrX	76411638	150611	G	GG	AAAA
rs28542821	A_20_P00200255	chrX	76637114	68271	NN	NN	NN
rs6623224	A_20_P00200258	chrX	77507589	113754	G	GA	GGGG
rs321011	A_20_P00302165	chrX	77622606	103956	NN	TG	TTGG
rs5913706	A_20_P00200260	chrX	77674407	18277	C	CC	CCTT
rs16979351	A_20_P00302168	chrX	78040730	12254	A	NN	NN
rs5912779	A_20_P00302171	chrX	78412492	817	T	TT	TTTT
rs5959289	A_20_P00302173	chrX	78547181	108733	C	NN	NN
rs6522917	A_20_P00302174	chrX	78557384	147957	NN	NN	NN
rs6619855	A_20_P00302175	chrX	78558037	100631	C	CC	NN
rs7051976	A_20_P00302177	chrX	78657892	128004	NN	NN	NN
rs12832235	A_20_P00200273	chrX	78672703	73472	NN	AA	AAAA
rs2205677	A_20_P00200274	chrX	78693408	139033	NN	TT	TTTT
rs5958902	A_20_P00302184	chrX	78816789	4047	A	AA	AAAA
rs1353455	A_20_P00302185	chrX	78847909	170958	A	AA	AAAA
rs7882388	A_20_P00302186	chrX	78852880	41038	C	NN	NN
rs12007174	A_20_P00200281	chrX	78854426	807	NN	NN	NN
rs1390532	A_20_P00302189	chrX	78912279	16817	G	TG	TTTT
rs6523288	A_20_P00302191	chrX	78983723	138925	C	CG	CCGG
rs4419949	A_20_P00200286	chrX	79000872	176492	NN	AG	AAGG
rs5959502	A_20_P00302193	chrX	79075155	10352	T	TT	TTCC
rs5958957	A_20_P00200288	chrX	79089159	50685	C	CC	CCCC
rs7882581	A_20_P00302196	chrX	79125677	27892	G	GG	GGGG
rs5958987	A_20_P00302199	chrX	79134167	121086	T	NN	NN
rs1594200	A_20_P00302200	chrX	79164827	92907	A	AC	AAAA
rs6621367	A_20_P00302201	chrX	79220877	13573	NN	GG	AAGG
rs6616360	A_20_P00302202	chrX	79221954	74368	G	NN	NN
rs2175647	A_20_P00200300	chrX	79902347	73139	T	CC	TTTT
rs6524030	A_20_P00302207	chrX	80126791	139093	NN	TG	TTTT
rs5959745	A_20_P00200302	chrX	80186605	121678	C	CG	CCGG

SNP ID	Probe Name	Chromosome	SNP Position	Feature Number	Father	Mother	Proband
rs5959759	A_20_P00200304	chrX	80212816	66354	C	CT	CCTT
rs5959843	A_20_P00302214	chrX	80464078	23261	NN	NN	NN
rs966360	A_20_P00302215	chrX	80577407	77985	G	AG	AAGG
rs6622466	A_20_P00200311	chrX	80652855	816	G	GA	GGAA
rs5913478	A_20_P00200312	chrX	80715901	118951	NN	GA	GGAA
rs1563086	A_20_P00200313	chrX	80723710	56330	T	NN	NN
rs2084976	A_20_P00200314	chrX	80730793	116397	T	TA	TTAA
rs5913526	A_20_P00302221	chrX	80808824	30215	NN	GA	GGAA
rs9988345	A_20_P00200316	chrX	80810438	27810	G	CG	CCGG
rs5913553	A_20_P00200317	chrX	80851722	95759	C	CT	CCTT
rs5913555	A_20_P00200318	chrX	80852406	145868	C	NN	NN
rs5967546	A_20_P00302231	chrX	81364855	127640	NN	AC	AACC
rs5969666	A_20_P00200328	chrX	81749901	16724	A	AA	NN
rs5969669	A_20_P00200329	chrX	81751322	23227	T	TT	TTCC
rs7063602	A_20_P00200330	chrX	81756638	64112	C	NN	NN
rs7890495	A_20_P00200334	chrX	82090076	163327	G	AA	AAAA
rs6616832	A_20_P00302242	chrX	82236967	154154	A	NN	NN
rs5922743	A_20_P00200337	chrX	82244908	74462	C	CC	CCCC
rs5968084	A_20_P00302244	chrX	82260787	148140	A	AA	AAAA
rs11092824	A_20_P00200340	chrX	82397781	5670	A	AA	AAAA
rs210446	A_20_P00302247	chrX	82421770	144572	NN	GT	GGGG
rs3904089	A_20_P00200344	chrX	82464602	73490	C	GC	GGCC
rs12007887	A_20_P00200345	chrX	82485316	144345	NN	TC	TTCC
rs4828230	A_20_P00302252	chrX	82613651	163375	G	AG	AAGG
rs2474852	A_20_P00302254	chrX	82835420	72491	G	GG	AAGG
rs1418933	A_20_P00200350	chrX	83494950	136215	NN	CC	TTTT
rs5923046	A_20_P00200354	chrX	83841510	112415	A	AC	AAAA
rs2341840	A_20_P00200355	chrX	83909789	33297	A	AA	AAGG
rs17324727	A_20_P00302264	chrX	84017262	25751	NN	NN	NN
rs12852750	A_20_P00200360	chrX	84092429	93291	A	NN	NN
rs4828301	A_20_P00302267	chrX	84136032	171432	NN	NN	NN
rs5923199	A_20_P00200362	chrX	84190601	173464	C	NN	NN
rs16980077	A_20_P00302269	chrX	84321646	27029	T	TT	TTTT
rs5968444	A_20_P00200364	chrX	84349446	111012	A	NN	NN
rs5968458	A_20_P00200365	chrX	84380619	96863	NN	AG	AAAA
rs17278401	A_20_P00200366	chrX	84532273	7624	C	CC	CCTT
rs12008480	A_20_P00302273	chrX	84534115	158848	T	TG	TTTT
rs6617059	A_20_P00302275	chrX	84691934	73711	A	AA	AACC
rs5923308	A_20_P00302277	chrX	84728816	37833	NN	NN	NN
rs4492518	A_20_P00302278	chrX	84737931	153864	NN	NN	CCCC
rs7051900	A_20_P00302279	chrX	84831312	91583	T	TT	TTTT
rs10126406	A_20_P00200374	chrX	84835549	113430	T	TT	TTTT
rs5967586	A_20_P00302284	chrX	84904756	100549	T	TT	CCCC
rs7879492	A_20_P00200380	chrX	85018625	108750	NN	NN	NN
rs5923385	A_20_P00200381	chrX	85048954	153725	G	GG	GGGG
rs5922198	A_20_P00302288	chrX	85056303	92148	G	GG	GGGG
rs5923408	A_20_P00200384	chrX	85200129	120812	G	NN	NN

SNP ID	Probe Name	Chromosome	SNP Position	Feature Number	Father	Mother	Proband
rs242857	A_20_P00302294	chrX	85431363	110858	C	TT	CCCC
rs6418342	A_20_P00200393	chrX	85613561	107192	C	CC	CCCC
rs6623702	A_20_P00200394	chrX	85797379	34578	G	GG	GGGG
rs5922256	A_20_P00302302	chrX	85806308	160169	G	NN	NN
rs12689011	A_20_P00200397	chrX	85870719	166001	A	AG	AAAA
rs5968962	A_20_P00302304	chrX	85915883	108116	A	AA	GGAA
rs7056464	A_20_P00200399	chrX	85960465	721	G	AG	GGGG
rs5923624	A_20_P00200400	chrX	85996514	75270	G	NN	NN
rs2040401	A_20_P00302311	chrX	86182600	140533	G	GG	GGCC
rs2187772	A_20_P00302312	chrX	86207212	98323	NN	NN	CCCC
rs2858668	A_20_P00302314	chrX	86234433	59563	A	AC	AAAA
rs6623907	A_20_P00200410	chrX	86399008	147382	C	CC	CCGG
rs5969110	A_20_P00200411	chrX	86425426	98616	C	TT	TTTT
rs5923865	A_20_P00200413	chrX	86433747	36235	T	TA	TTAA
rs5923874	A_20_P00302320	chrX	86439877	71847	A	TA	TTAA
rs2176391	A_20_P00200417	chrX	86509673	19731	NN	NN	NN
rs503848	A_20_P00200419	chrX	86599254	26631	G	AG	NN
rs10521379	A_20_P00200420	chrX	86667493	134191	NN	NN	NN
rs5922442	A_20_P00302327	chrX	86672042	133883	C	TT	NN
rs5922443	A_20_P00200422	chrX	86672096	119378	C	TT	TTCC
rs2051621	A_20_P00302330	chrX	86741400	165919	C	CT	NN
rs34380497	A_20_P00302331	chrX	86774014	98535	C	TT	NN
rs6652458	A_20_P00302332	chrX	86818343	46047	C	CC	CCCC
rs6617435	A_20_P00200427	chrX	86818655	11552	C	AA	AACC
rs5922463	A_20_P00200428	chrX	86862721	34101	A	NN	NN
rs11092976	A_20_P00200429	chrX	86929178	66550	C	CC	CCCC
rs5924115	A_20_P00302337	chrX	87009043	57589	T	TT	TTTT
rs5922496	A_20_P00200434	chrX	87120891	119159	NN	AA	AAAA
rs12838133	A_20_P00200435	chrX	87127557	166573	NN	CC	CCCC
rs12689405	A_20_P00200436	chrX	87127607	72905	NN	TT	TTTT
rs12010011	A_20_P00200437	chrX	87170171	137103	G	GG	GGGG
rs12011713	A_20_P00200438	chrX	87315816	177712	A	AA	AAAA
rs5924214	A_20_P00200439	chrX	87389022	14829	G	NN	GGGG
rs12389971	A_20_P00200441	chrX	87416995	110632	C	CC	CCCC
rs5969500	A_20_P00200442	chrX	87451481	15140	T	TG	TTTT
rs7052428	A_20_P00200443	chrX	87483357	176369	C	CC	CCCC
rs7884996	A_20_P00302350	chrX	87502227	84296	C	CC	TTTT
rs5969623	A_20_P00200445	chrX	87539700	27056	C	CC	CCCC
rs4240071	A_20_P00302355	chrX	87628131	37152	C	CC	CCCC
rs12834358	A_20_P00302356	chrX	87628544	78614	A	AA	AAAA
rs6617592	A_20_P00200451	chrX	87632419	41898	T	TT	CCCC
rs5020863	A_20_P00200452	chrX	87634843	59922	NN	CC	AAAA
rs6614771	A_20_P00302359	chrX	87676893	88330	G	AG	AAAA
rs6617674	A_20_P00302360	chrX	87731813	139407	G	GG	AAAA
rs6614803	A_20_P00200456	chrX	87759164	73710	G	TG	GGGG
rs7881387	A_20_P00302364	chrX	87981878	49797	A	AG	AAGG
rs17003522	A_20_P00200459	chrX	88006784	38802	C	NN	CCCC

SNP ID	Probe Name	Chromosome	SNP Position	Feature Number	Father	Mother	Proband
rs12013021	A_20_P00302366	chrX	88121025	120010	NN	NN	NN
rs847465	A_20_P00302368	chrX	88133267	49205	A	GA	GGAA
rs6614847	A_20_P00302369	chrX	88165587	59525	C	CC	NN
rs5985024	A_20_P00200464	chrX	88180017	57892	NN	GG	GGGG
rs12395018	A_20_P00302372	chrX	88260812	8824	A	AA	AAGG
rs6617812	A_20_P00200467	chrX	88319297	5369	G	GG	AAGG
rs2400323	A_20_P00200468	chrX	88364371	114076	A	AA	GGAA
rs5941136	A_20_P00302375	chrX	88368893	125764	NN	NN	GGGG
rs5942412	A_20_P00200470	chrX	88392517	126168	T	TT	TTTT
rs11093199	A_20_P00200478	chrX	89230634	80037	G	AA	AAAA
rs609835	A_20_P00200487	chrX	90549341	161713	G	NN	NN
rs637227	A_20_P00302395	chrX	90566657	141186	NN	GG	AAAA
rs503416	A_20_P00200491	chrX	90572926	59353	NN	GG	AAAA
rs555029	A_20_P00200492	chrX	90585859	153036	C	CC	TTTT
rs7064930	A_20_P00200493	chrX	90618824	98079	T	TT	TTTT
rs6615243	A_20_P00302401	chrX	90811700	92169	G	GG	GGGG
rs4568761	A_20_P00200498	chrX	91439067	166398	NN	NN	NN
rs4141535	A_20_P00200499	chrX	91454022	91268	C	CC	GGGG
rs5984161	A_20_P00302406	chrX	91476337	145669	G	AG	AAAA
rs5984918	A_20_P00302410	chrX	91526734	122094	A	AT	AAAA
rs12007684	A_20_P00302411	chrX	91551621	118316	NN	NN	NN
rs1209373	A_20_P00200511	chrX	92472075	117536	A	AA	AAAA
rs1402080	A_20_P00200512	chrX	92517765	65472	A	NN	NN
rs5983478	A_20_P00302421	chrX	92605644	133322	T	NN	NN
rs1356849	A_20_P00200516	chrX	92606749	34715	C	CC	TTCC
rs4524969	A_20_P00302423	chrX	92621780	49157	NN	TT	TTCC
rs5983497	A_20_P00302424	chrX	92635403	10659	NN	NN	TTTT
rs6615524	A_20_P00200519	chrX	92698181	70376	T	NN	NN
rs1326184	A_20_P00200520	chrX	92708367	83800	C	TC	TTTT
rs5983223	A_20_P00200521	chrX	92710457	70217	NN	GT	TTTT
rs1326180	A_20_P00200522	chrX	92742104	177762	NN	AT	AAAA
rs1575859	A_20_P00302429	chrX	92864021	134333	G	NN	NN
rs12008360	A_20_P00200525	chrX	92953946	43851	T	TT	TTCC
rs16983122	A_20_P00200526	chrX	92998227	87744	G	GG	GGGG
rs5983616	A_20_P00302433	chrX	93001457	106922	T	TC	TTCC
rs1207445	A_20_P00200528	chrX	93024937	96768	C	TT	TTCC
rs7061368	A_20_P00200529	chrX	93103836	101223	T	NN	NN
rs5983154	A_20_P00200530	chrX	93224356	84685	NN	GG	GGGG
rs7879599	A_20_P00200531	chrX	93289323	2487	NN	NN	NN
rs6619481	A_20_P00200534	chrX	93342649	135052	NN	NN	NN
rs1342188	A_20_P00200535	chrX	93346968	87250	NN	NN	NN
rs5939888	A_20_P00200536	chrX	93349228	18125	T	NN	NN
rs4892941	A_20_P00302443	chrX	93351241	20448	G	AA	GGGG
rs5983343	A_20_P00200538	chrX	93351801	139661	G	AA	AAGG
rs708555	A_20_P00302445	chrX	93384125	3041	T	TT	NN
rs17276081	A_20_P00200540	chrX	93413622	33446	T	CC	TTTT
rs808746	A_20_P00302447	chrX	93430407	86053	G	GG	CCCC

SNP ID	Probe Name	Chromosome	SNP Position	Feature Number	Father	Mother	Proband
rs808754	A_20_P00302448	chrX	93438034	160031	G	GG	GGGG
rs213730	A_20_P00302449	chrX	93467700	48873	T	NN	NN
rs5939930	A_20_P00200545	chrX	93506285	138171	NN	CC	NN
rs1599025	A_20_P00200546	chrX	93512105	175053	A	AG	GGGG
rs5990783	A_20_P00200548	chrX	93565396	19833	G	GG	GGGG
rs16981623	A_20_P00302457	chrX	93629407	50546	C	CC	CCCC
rs4969671	A_20_P00302460	chrX	93772955	126216	NN	GG	AAAA
rs12845726	A_20_P00302461	chrX	93845375	20077	NN	AG	AAGG
rs3120900	A_20_P00302462	chrX	94010176	140414	G	GG	NN
rs3130079	A_20_P00200557	chrX	94014903	167804	T	TT	NN
rs7057800	A_20_P00302464	chrX	94061017	171768	NN	AA	AAAA
rs4631631	A_20_P00200560	chrX	94240799	114268	NN	AG	AAAA
rs5990712	A_20_P00302467	chrX	94254310	113244	A	NN	NN
rs2883235	A_20_P00200562	chrX	94272863	37339	NN	TC	TTTT
rs5990264	A_20_P00200564	chrX	94314713	34473	A	NN	NN
rs5950193	A_20_P00200567	chrX	94381450	27461	A	NN	NN
rs5990333	A_20_P00200571	chrX	94745550	160373	T	TT	AAAA
rs5990338	A_20_P00200572	chrX	94757342	128594	G	NN	GGGG
rs5990386	A_20_P00302479	chrX	94911723	52256	T	TT	TTCC
rs2225037	A_20_P00200576	chrX	95062716	27883	T	TC	TTCC
rs4142316	A_20_P00200577	chrX	95187179	120987	G	AA	AAGG
rs12390413	A_20_P00200578	chrX	95189792	17309	NN	NN	NN
rs1951848	A_20_P00302485	chrX	95254089	14559	C	CC	TTTT
rs5949360	A_20_P00302486	chrX	95272712	119808	NN	NN	NN
rs5949366	A_20_P00302487	chrX	95294529	80315	G	AG	AAAA
rs5949789	A_20_P00200583	chrX	95353643	15795	NN	GA	GGGG
rs9887355	A_20_P00200586	chrX	95579713	140960	A	AA	GGGG
rs5990546	A_20_P00200587	chrX	95612086	122488	G	GG	GGGG
rs2316984	A_20_P00302494	chrX	95647639	121299	NN	NN	NN
rs17337750	A_20_P00302498	chrX	95796116	141236	NN	TC	TTCC
rs17333633	A_20_P00200593	chrX	95796179	43581	G	CG	CCGG
rs7054746	A_20_P00302504	chrX	95917551	68017	T	TT	TTTT
rs6620146	A_20_P00302506	chrX	95996045	52755	T	TC	CCCC
rs4969602	A_20_P00200602	chrX	96003293	72036	A	AC	CCCC
rs12013445	A_20_P00200603	chrX	96016517	84000	G	NN	NN
rs6523058	A_20_P00200604	chrX	96024038	88081	T	TC	TTCC
rs384448	A_20_P00302511	chrX	96047519	118949	NN	CC	GGCC
rs233221	A_20_P00200606	chrX	96097770	35606	A	AA	GGAA
rs5921005	A_20_P00302513	chrX	96221116	180323	A	AG	NN
rs6523069	A_20_P00200608	chrX	96289388	39624	A	AA	AATT
rs5921281	A_20_P00200609	chrX	96319939	101162	T	CC	TTCC
rs6620228	A_20_P00302516	chrX	96416258	46058	C	GG	CCCC
rs5921766	A_20_P00302517	chrX	96535965	123586	NN	GG	NN
rs6620269	A_20_P00200612	chrX	96648294	80676	A	AC	CCCC
rs2353564	A_20_P00200613	chrX	96678192	79966	C	CG	NN
rs2027240	A_20_P00200615	chrX	96791827	62197	A	AA	AAAA
rs5967348	A_20_P00302522	chrX	96948111	97571	A	AA	AAAA

SNP ID	Probe Name	Chromosome	SNP Position	Feature Number	Father	Mother	Proband
rs900011	A_20_P00302524	chrX	96989691	178953	C	CC	NN
rs2497877	A_20_P00302526	chrX	96993229	14524	NN	NN	CCCC
rs1383650	A_20_P00302527	chrX	97001284	134125	G	GG	NN
rs1458301	A_20_P00302528	chrX	97113336	138921	NN	NN	NN
rs7877389	A_20_P00302529	chrX	97197534	78514	G	GG	AAAA
rs319638	A_20_P00302530	chrX	97217415	116125	A	AG	AAGG
rs815641	A_20_P00302531	chrX	97219791	52039	C	CT	CCTT
rs1152064	A_20_P00302533	chrX	97327785	88829	C	CC	CCGG
rs1380027	A_20_P00302536	chrX	97395995	77663	G	GG	CCCC
rs4400520	A_20_P00302537	chrX	97600336	12937	A	NN	AAAA
rs17322499	A_20_P00200632	chrX	97600779	50147	NN	CC	GGGG
rs2038888	A_20_P00200634	chrX	97655951	111828	T	CT	CCTT
rs7876198	A_20_P00302541	chrX	97661823	69192	T	TT	TTGG
rs1886826	A_20_P00200636	chrX	97665910	90199	C	GC	GGGG
rs6616002	A_20_P00200638	chrX	97690232	158540	A	CA	AAAA
rs5921202	A_20_P00200642	chrX	98016068	171552	C	CC	GGGG
rs7064493	A_20_P00200643	chrX	98052552	109428	C	NN	NN
rs2478362	A_20_P00200645	chrX	98111834	86645	C	CC	TTCC
rs5920723	A_20_P00200650	chrX	98390248	136642	T	TT	TTTT
rs7881142	A_20_P00200651	chrX	98438386	165473	C	CC	CCTT
rs5920729	A_20_P00200652	chrX	98446580	137648	T	NN	TTTT
rs6652783	A_20_P00200658	chrX	98763231	29184	G	GG	GGCC
rs16983166	A_20_P00302566	chrX	98961612	179947	A	AA	AAAA
rs2177702	A_20_P00200661	chrX	98968833	59707	C	CC	NN
rs1924232	A_20_P00302568	chrX	98987458	103271	NN	NN	AAAA
rs5921393	A_20_P00200664	chrX	99098756	51401	T	TT	TTTT
rs974308	A_20_P00302571	chrX	99161829	109035	A	AA	GGGG
rs7880875	A_20_P00200666	chrX	99164518	94528	G	NN	NN
rs2085984	A_20_P00200667	chrX	99223778	25831	C	CC	NN
rs4827982	A_20_P00200668	chrX	99242490	103248	NN	NN	CCCC
rs5921491	A_20_P00200670	chrX	99380931	73043	NN	AA	AAAA
rs7064056	A_20_P00302577	chrX	99567435	49163	T	TT	TTTT
rs5966696	A_20_P00200672	chrX	99604645	157986	NN	NN	NN
rs5967134	A_20_P00200673	chrX	99684420	166072	NN	NN	NN
rs2073162	A_20_P00302580	chrX	99849017	104721	A	GA	GGGG
rs1204396	A_20_P00302581	chrX	99882199	157790	NN	NN	NN
rs4828040	A_20_P00302584	chrX	99928949	23672	T	TT	NN
rs1794474	A_20_P00302587	chrX	100041336	70326	T	TT	TTTT
rs2858263	A_20_P00200682	chrX	100063362	132014	G	NN	NN
rs11798428	A_20_P00200685	chrX	100252108	95151	NN	CC	CCCC
rs5921710	A_20_P00200687	chrX	100278109	78153	T	TT	CCCC
rs4827886	A_20_P00302595	chrX	100308635	76341	NN	NN	NN
rs6652369	A_20_P00200690	chrX	100337603	102824	NN	NN	TTTT
rs7883144	A_20_P00302598	chrX	100387337	81308	C	NN	NN
rs6523455	A_20_P00302600	chrX	100410747	71213	A	AA	NN
rs1054490	A_20_P00200695	chrX	100418447	63174	A	GG	AAAA
rs5921744	A_20_P00200696	chrX	100445964	152559	T	CC	TTCC

SNP ID	Probe Name	Chromosome	SNP Position	Feature Number	Father	Mother	Proband
rs5967282	A_20_P00302604	chrX	100515332	107994	NN	NN	NN
rs1475972	A_20_P00302605	chrX	100518165	123205	NN	NN	NN
rs6621074	A_20_P00200703	chrX	100730446	107363	NN	CC	CCCC
rs963618	A_20_P00302610	chrX	100743037	11181	T	CC	TTTT
rs6418213	A_20_P00200705	chrX	101018201	159449	C	CC	AAAA
rs5945091	A_20_P00302614	chrX	101106502	70128	NN	AA	NN
rs16984503	A_20_P00200710	chrX	101211781	46019	C	CC	TTTT
rs5944855	A_20_P00302620	chrX	101378086	60279	NN	TT	GGGG
rs5987637	A_20_P00200715	chrX	102092440	147960	C	TC	TTTT
rs7054894	A_20_P00302622	chrX	102160785	84298	A	AA	GGGG
rs5987654	A_20_P00200719	chrX	102247987	121344	A	AA	NN
rs5987510	A_20_P00302628	chrX	102430992	124640	C	CC	NN
rs11092430	A_20_P00302629	chrX	102476263	174007	NN	CC	TTTT
rs17342518	A_20_P00200724	chrX	102503819	13997	NN	NN	NN
rs387960	A_20_P00200725	chrX	102519583	164010	C	CC	CCCC
rs2756053	A_20_P00200726	chrX	102546049	176572	NN	TG	TTTT
rs5987722	A_20_P00302633	chrX	102561270	60536	NN	GG	GGGG
rs568707	A_20_P00200730	chrX	103007196	122479	NN	NN	NN
rs2385048	A_20_P00302637	chrX	103010109	52387	C	GC	GGGG
rs5945837	A_20_P00302640	chrX	103105062	6134	NN	NN	NN
rs2022466	A_20_P00302641	chrX	103114077	172463	C	CA	CCAA
rs5916952	A_20_P00302645	chrX	103449423	21619	NN	TT	TTTT
rs7050692	A_20_P00302647	chrX	103518530	19544	C	CC	GGGG
rs6621809	A_20_P00302648	chrX	103557686	162670	NN	GT	GGTT
rs1983803	A_20_P00200745	chrX	103730287	148883	C	TC	TTTT
rs7058613	A_20_P00302652	chrX	103751083	31018	T	TG	TTGG
rs5917103	A_20_P00302653	chrX	103838495	92078	A	CC	CCAA
rs1015505	A_20_P00302655	chrX	103910924	51598	T	TT	TTCC
rs1540291	A_20_P00302657	chrX	104205248	53011	C	GG	GGGG
rs5917182	A_20_P00200752	chrX	104211906	179117	G	AA	AAGG
rs1034402	A_20_P00302659	chrX	104233978	109865	NN	GG	NN
rs988176	A_20_P00200754	chrX	104237750	25821	T	CC	CCTT
rs7876119	A_20_P00200756	chrX	104572607	3284	A	AA	AATT
rs1321379	A_20_P00200758	chrX	104633932	130536	NN	NN	NN
rs5962517	A_20_P00200759	chrX	104670809	52558	A	AA	AAAA
rs16984773	A_20_P00200760	chrX	104676299	102397	G	GG	GGGG
rs5916922	A_20_P00200763	chrX	104785859	45926	A	AA	AAAA
rs5962589	A_20_P00302671	chrX	105068195	132323	G	NN	GGGG
rs209106	A_20_P00302672	chrX	105233536	166384	NN	AA	AAAA
rs11796132	A_20_P00302674	chrX	105299374	52219	G	NN	GGGG
rs2754830	A_20_P00302677	chrX	105466607	37618	A	CA	CCCC
rs12014793	A_20_P00200774	chrX	105892783	20857	NN	NN	CCCC
rs1582802	A_20_P00302681	chrX	105900701	93377	A	AA	AAAA
rs7881669	A_20_P00302682	chrX	105904946	65339	NN	TT	NN
rs5962726	A_20_P00200777	chrX	105948275	30252	NN	NN	GGGG
rs5962360	A_20_P00200778	chrX	105957674	64893	G	GG	GGGG
rs12008279	A_20_P00200780	chrX	106160702	41623	G	NN	NN

SNP ID	Probe Name	Chromosome	SNP Position	Feature Number	Father	Mother	Proband
rs6616619	A_20_P00302687	chrX	106276933	12069	NN	NN	NN
rs980098	A_20_P00302688	chrX	106311924	92985	NN	NN	NN
rs6523924	A_20_P00302689	chrX	106385336	10675	T	NN	NN
rs1285575	A_20_P00302690	chrX	106413956	80079	G	NN	GGGG
rs16985154	A_20_P00302692	chrX	106555611	161252	C	CC	CCCC
rs16985203	A_20_P00200787	chrX	106741642	104014	T	TT	TTTT
rs5962866	A_20_P00200788	chrX	106868974	131186	G	GG	GGGG
rs5962410	A_20_P00200790	chrX	107073048	159783	C	CC	CCCC
rs807185	A_20_P00302699	chrX	107340718	51574	A	AA	TTAA
rs5973822	A_20_P00200794	chrX	107396958	110473	A	AA	AAAA
rs1266763	A_20_P00302703	chrX	107508355	81968	G	GG	GGGG
rs5973878	A_20_P00302705	chrX	107715484	62641	G	TT	TTTT
rs28843178	A_20_P00200800	chrX	107777048	85113	G	AA	AAAA
rs28469248	A_20_P00302707	chrX	107833524	165697	NN	NN	NN
rs12847663	A_20_P00302709	chrX	108020989	139225	NN	AG	AAAA
rs6567831	A_20_P00302711	chrX	108167569	55577	G	GA	AAAA
rs563074	A_20_P00302720	chrX	108617053	39363	C	CC	AAAA
rs2300116	A_20_P00302722	chrX	108702640	93236	G	GG	GGGG
rs1547612	A_20_P00200819	chrX	108823401	177608	G	GG	GGGG
rs861431	A_20_P00200820	chrX	108832955	151320	T	TT	TTTT
rs5943427	A_20_P00302727	chrX	108973339	21208	G	GG	GGCC
rs7885908	A_20_P00200826	chrX	109071961	94905	NN	NN	NN
rs2475823	A_20_P00200828	chrX	109159248	157360	NN	NN	NN
rs16985896	A_20_P00302736	chrX	109181454	14444	A	NN	NN
rs2214190	A_20_P00200833	chrX	109209433	135737	NN	CC	CCGG
rs5985446	A_20_P00200834	chrX	109365260	11739	NN	NN	NN
rs760953	A_20_P00200835	chrX	109609999	55597	NN	NN	NN
rs6567865	A_20_P00302742	chrX	109701864	7174	C	CC	CCCC
rs5942958	A_20_P00200838	chrX	109784640	133694	NN	AA	AAAA
rs5942648	A_20_P00200839	chrX	109788727	121471	G	GG	AAAA
rs5943055	A_20_P00200841	chrX	109935846	126078	G	GG	AAAA
rs4298660	A_20_P00200844	chrX	110130584	78066	NN	NN	NN
rs5943076	A_20_P00200845	chrX	110139805	82003	NN	NN	NN
rs5942700	A_20_P00200846	chrX	110158676	145846	NN	NN	NN
rs5985312	A_20_P00200847	chrX	110194225	163177	NN	GG	GGGG
rs5942708	A_20_P00200848	chrX	110236985	138613	NN	NN	CCCC
rs16986325	A_20_P00200850	chrX	110296238	111991	C	CC	CCCC
rs16986378	A_20_P00302758	chrX	110380340	65665	NN	NN	NN
rs7879095	A_20_P00200853	chrX	110405884	141355	G	GG	GGGG
rs12013711	A_20_P00302760	chrX	110494841	42109	C	CC	GGGG
rs7050821	A_20_P00200857	chrX	110726064	59982	T	TT	TTTT
rs5985641	A_20_P00200859	chrX	110984432	171790	NN	GG	GGGG
rs3027740	A_20_P00302767	chrX	111067949	152432	C	CC	TTTT
rs767034	A_20_P00200862	chrX	111168829	16633	C	CC	TTTT
rs7887095	A_20_P00200863	chrX	111199462	167479	G	GG	GGGG
rs7887124	A_20_P00302770	chrX	111199520	103101	G	NN	NN
rs17222629	A_20_P00302771	chrX	111215692	103934	T	TT	TTTT

SNP ID	Probe Name	Chromosome	SNP Position	Feature Number	Father	Mother	Proband
rs16986729	A_20_P00302772	chrX	111249119	180163	T	TT	TTTT
rs12009375	A_20_P00302775	chrX	111369245	158219	NN	NN	NN
rs6568005	A_20_P00200870	chrX	111372933	144671	G	GG	GGGG
rs7066610	A_20_P00200871	chrX	111510074	71440	G	GG	GGGG
rs7473376	A_20_P00302779	chrX	111647643	64448	NN	CC	CCCC
rs7473003	A_20_P00200876	chrX	111686263	127663	G	AA	AAAA
rs5973960	A_20_P00302785	chrX	111762314	148868	G	GG	GGGG
rs5974281	A_20_P00200880	chrX	111772603	169525	A	GG	AAAA
rs5974256	A_20_P00200883	chrX	111909907	140419	T	TT	TTTT
rs16987057	A_20_P00200884	chrX	111929652	124755	C	CC	CCCC
rs7891953	A_20_P00302791	chrX	111932727	67174	NN	CC	CCCC
rs5929222	A_20_P00200886	chrX	111961559	120793	A	AA	AAAA
rs16987070	A_20_P00200887	chrX	111977297	84330	C	NN	NN
rs16987077	A_20_P00200888	chrX	111982986	30881	G	GG	GGGG
rs687956	A_20_P00200889	chrX	112080302	56780	A	AA	AAAA
rs646398	A_20_P00200890	chrX	112107726	82729	A	AA	GGGG
rs5974298	A_20_P00200891	chrX	112114927	89200	G	GG	GGGG
rs4829511	A_20_P00302798	chrX	112119452	143474	A	AA	GGGG
rs7055219	A_20_P00200894	chrX	112195181	80252	T	TT	TTTT
rs2213995	A_20_P00200895	chrX	112216628	46016	A	GA	GGGG
rs3125995	A_20_P00302802	chrX	112299829	21685	A	AC	AAAA
rs6643198	A_20_P00302805	chrX	112405177	175923	T	NN	NN
rs3125982	A_20_P00200900	chrX	112406536	125493	C	NN	NN
rs6643204	A_20_P00200901	chrX	112409948	162827	NN	AG	GGGG
rs5974006	A_20_P00200902	chrX	112413313	2427	G	GG	GGGG
rs5929629	A_20_P00200907	chrX	112705652	148048	T	NN	NN
rs16987325	A_20_P00200909	chrX	112717391	105111	T	TT	CCCC
rs7050224	A_20_P00302817	chrX	112823835	115229	A	AA	GGGG
rs5929366	A_20_P00302818	chrX	112893690	119118	NN	NN	NN
rs2887562	A_20_P00302819	chrX	112926278	47689	NN	NN	NN
rs2905259	A_20_P00200915	chrX	113013817	126638	C	NN	NN
rs2905249	A_20_P00200916	chrX	113022540	139114	T	TT	TTTT
rs3007187	A_20_P00200918	chrX	113159820	35569	G	NN	NN
rs6568218	A_20_P00200919	chrX	113201276	78639	NN	CC	CCGG
rs5974199	A_20_P00200920	chrX	113234397	38939	A	AG	AAGG
rs5974208	A_20_P00200921	chrX	113256582	154563	A	AA	AACC
rs13328639	A_20_P00200923	chrX	113357739	175352	A	AA	AAAA
rs2188139	A_20_P00302831	chrX	113424089	176482	A	AA	GGGG
rs9779655	A_20_P00302832	chrX	113457413	76276	G	GG	GGGG
rs12388307	A_20_P00200927	chrX	113458520	135838	T	TT	TTTT
rs11152812	A_20_P00200928	chrX	113473495	55578	C	CC	TTCC
rs12390894	A_20_P00200929	chrX	113495223	12027	C	CC	TTTT
rs5988067	A_20_P00302836	chrX	113592940	128919	NN	GG	CCCC
rs5987853	A_20_P00302837	chrX	113643472	25506	NN	GC	CCCC
rs12008899	A_20_P00200933	chrX	113677159	166560	G	AG	AAAA
rs5987802	A_20_P00200937	chrX	113931419	41073	NN	AA	AAAA
rs5987819	A_20_P00302845	chrX	114007882	33975	A	AA	GGGG

SNP ID	Probe Name	Chromosome	SNP Position	Feature Number	Father	Mother	Proband
rs5946009	A_20_P00302846	chrX	114086293	61429	NN	AA	GGGG
rs1360851	A_20_P00302848	chrX	114140485	45855	T	NN	NN
rs5946230	A_20_P00302849	chrX	114152227	99372	NN	GG	AAAA
rs17095760	A_20_P00302850	chrX	114213384	146656	C	CC	CCCC
rs17095780	A_20_P00200945	chrX	114218562	165501	A	AA	AAGG
rs5988201	A_20_P00302853	chrX	114260915	163535	C	CC	GGCC
rs5946042	A_20_P00200948	chrX	114385014	81314	NN	AA	AAAA
rs6644208	A_20_P00200950	chrX	114422214	152887	A	AA	AAAA
rs17326555	A_20_P00200952	chrX	114646245	97168	C	CC	CCCC
rs2207050	A_20_P00200953	chrX	114657765	28948	C	CC	CCCC
rs6643974	A_20_P00302862	chrX	114757602	33773	G	AA	GGGG
rs871774	A_20_P00302864	chrX	114880423	76636	T	TT	CCCC
rs5987981	A_20_P00302865	chrX	114909139	30689	C	CC	CCCC
rs9698964	A_20_P00302867	chrX	115169062	103473	A	AA	AAAA
rs6608553	A_20_P00302868	chrX	115194764	76886	G	GG	AAAA
rs5991066	A_20_P00200963	chrX	115202717	91105	NN	TT	CCCC
rs5991067	A_20_P00200964	chrX	115206378	99392	NN	TT	TTTT
rs7060450	A_20_P00200968	chrX	115353791	87645	NN	AA	AAAA
rs2001124	A_20_P00200976	chrX	115622273	84491	A	NN	NN
rs4824326	A_20_P00200977	chrX	115622796	112452	T	CT	CCCC
rs12384658	A_20_P00302884	chrX	115740289	35948	G	GG	TTTT
rs5910306	A_20_P00302888	chrX	115960293	153477	NN	TC	CCCC
rs4549940	A_20_P00200983	chrX	115977337	141105	NN	CC	CCCC
rs4523445	A_20_P00302890	chrX	116022458	152957	A	AA	AAAA
rs4520345	A_20_P00200985	chrX	116027486	73485	A	AA	NN
rs5910924	A_20_P00302894	chrX	116194582	15580	A	AA	GGGG
rs4472692	A_20_P00302895	chrX	116205497	134168	C	TC	CCCC
rs4825734	A_20_P00302897	chrX	116219154	11026	A	CC	CCCC
rs5909793	A_20_P00302898	chrX	116238776	69815	NN	NN	NN
rs2078802	A_20_P00200993	chrX	116310686	36825	C	CT	CCTT
rs2819651	A_20_P00200995	chrX	116367458	2681	G	GG	NN
rs4825819	A_20_P00200996	chrX	116419723	154503	G	NN	NN
rs7888227	A_20_P00200997	chrX	116452943	84888	C	CC	TTTT
rs7052570	A_20_P00200998	chrX	116483612	111586	G	NN	GGGG
rs5956581	A_20_P00302907	chrX	116515049	63919	NN	AA	TTAA
rs5958342	A_20_P00201002	chrX	116516718	97428	A	NN	AAAA
rs5911818	A_20_P00201007	chrX	116562782	139239	NN	TT	CCCC
rs5911853	A_20_P00302914	chrX	116570975	161880	G	AA	AAGG
rs2040959	A_20_P00302915	chrX	116587448	155972	C	CC	TTTT
rs2041532	A_20_P00201011	chrX	116657938	74388	NN	NN	NN
rs5956745	A_20_P00201012	chrX	116662226	96703	NN	NN	NN
rs5958743	A_20_P00201014	chrX	116768614	168355	G	GG	GGGG
rs5912072	A_20_P00201015	chrX	116788806	178561	A	GG	GGAA
rs5910185	A_20_P00201016	chrX	116797444	144687	G	AA	AAAA
rs5910186	A_20_P00302923	chrX	116798070	136985	NN	CC	CCCC
rs6645853	A_20_P00201018	chrX	116809479	34885	NN	GG	GGGG
rs1860996	A_20_P00302928	chrX	116999372	18490	G	NN	GGGG

SNP ID	Probe Name	Chromosome	SNP Position	Feature Number	Father	Mother	Proband
rs6645982	A_20_P00302929	chrX	117031161	178701	T	TT	NN
rs10465419	A_20_P00302930	chrX	117043066	18514	G	GG	GGGG
rs7050298	A_20_P00302932	chrX	117239499	56667	G	GG	GGGG
rs12396515	A_20_P00302933	chrX	117269582	140667	NN	TT	TTTT
rs5956884	A_20_P00302936	chrX	117285716	38509	A	AA	GGGG
rs1230821	A_20_P00201031	chrX	117307726	22222	A	AA	AAAA
rs1230826	A_20_P00201032	chrX	117310280	176049	C	CC	CCCC
rs2154310	A_20_P00201035	chrX	117354626	91228	G	NN	NN
rs17317988	A_20_P00201036	chrX	117363908	151363	G	NN	NN
rs7052298	A_20_P00201037	chrX	117364722	74547	NN	NN	NN
rs1937929	A_20_P00201040	chrX	117443591	42389	T	TT	GGGG
rs5909532	A_20_P00201041	chrX	117444473	85955	T	TT	AAAA
rs5910339	A_20_P00302948	chrX	117463091	52411	C	CC	CCCC
rs6646165	A_20_P00201043	chrX	117475396	124117	G	GG	NN
rs5956960	A_20_P00201045	chrX	117514641	180502	T	TT	NN
rs17271460	A_20_P00201046	chrX	117542200	105416	A	NN	NN
rs6603412	A_20_P00302953	chrX	117572236	84448	NN	AA	AAAA
rs1294790	A_20_P00302954	chrX	117622214	17345	A	AG	AAAA
rs4825587	A_20_P00201049	chrX	117647745	99192	G	GG	CCCC
rs5957024	A_20_P00302956	chrX	117683897	104772	G	NN	NN
rs5956070	A_20_P00201052	chrX	117723426	64131	NN	NN	NN
rs5910394	A_20_P00302959	chrX	117742356	120991	T	TT	CCCC
rs5956071	A_20_P00201054	chrX	117745333	117783	T	NN	NN
rs1287166	A_20_P00201055	chrX	117770656	162492	G	GG	TTTT
rs1287173	A_20_P00201056	chrX	117774334	48638	G	GG	AAAA
rs2379114	A_20_P00302963	chrX	117797282	86545	NN	GA	NN
rs12014885	A_20_P00302964	chrX	117811446	32854	C	CC	CCCC
rs4825603	A_20_P00302965	chrX	117843867	90133	NN	GC	GGGG
rs261693	A_20_P00201061	chrX	117998682	120773	NN	NN	NN
rs1455862	A_20_P00302971	chrX	118135923	87252	NN	TT	TTTT
rs7883690	A_20_P00201066	chrX	118150016	22346	T	TC	TTCC
rs7056710	A_20_P00201068	chrX	118243780	17080	NN	CC	CCCC
rs5910531	A_20_P00302977	chrX	118306475	98	G	AA	GGGG
rs5957131	A_20_P00201072	chrX	118314276	83853	T	TT	CCCC
rs2499041	A_20_P00302981	chrX	118376853	76193	T	TT	GGGG
rs10521686	A_20_P00302982	chrX	118460470	46950	T	TT	CCCC
rs6603506	A_20_P00302984	chrX	118552412	62279	G	NN	NN
rs5957195	A_20_P00201081	chrX	118776063	78883	T	TT	NN
rs6646438	A_20_P00201082	chrX	118780463	50583	C	CC	CCGG
rs17327025	A_20_P00302990	chrX	118862545	66888	NN	CC	CCTT
rs2782212	A_20_P00302991	chrX	118873350	128157	NN	NN	NN
rs12835475	A_20_P00201086	chrX	118879208	146564	C	CC	GGCC
rs5910689	A_20_P00201087	chrX	118991537	169618	NN	GG	GGGG
rs1476663	A_20_P00302994	chrX	119105456	119433	NN	NN	NN
rs12116119	A_20_P00302998	chrX	119406993	148009	A	GG	GGGG
rs17336153	A_20_P00302999	chrX	119446675	20800	G	NN	NN
rs5957386	A_20_P00303000	chrX	119571304	7760	A	AA	AAAA

SNP ID	Probe Name	Chromosome	SNP Position	Feature Number	Father	Mother	Proband
rs5956219	A_20_P00303003	chrX	119617710	97930	NN	NN	NN
rs5910975	A_20_P00201098	chrX	119838025	144651	G	CC	GGGG
rs5910984	A_20_P00201099	chrX	119865020	122374	G	AG	AAAA
rs6645685	A_20_P00201102	chrX	119974283	80885	C	CG	CCCC
rs5911054	A_20_P00303011	chrX	120143772	7222	NN	AG	GGGG
rs5909801	A_20_P00201108	chrX	120296989	80714	NN	AG	AAAA
rs5957548	A_20_P00303015	chrX	120303963	46083	NN	TT	TTTT
rs17324121	A_20_P00303018	chrX	120320001	151029	NN	AA	AAAA
rs5957641	A_20_P00303021	chrX	120446215	13567	NN	TC	CCCC
rs5956302	A_20_P00201116	chrX	120450626	67196	G	GG	NN
rs5957652	A_20_P00303023	chrX	120457971	41499	A	TA	TTAA
rs7889558	A_20_P00303026	chrX	120728555	14181	A	AA	AAGG
rs5956347	A_20_P00303027	chrX	120737733	88509	NN	GG	AAGG
rs887504	A_20_P00201122	chrX	120789592	28502	G	GG	TTGG
rs2507236	A_20_P00201123	chrX	120818146	39806	T	TT	TTTT
rs12397099	A_20_P00201125	chrX	120976997	104668	NN	NN	NN
rs2185658	A_20_P00201128	chrX	121020284	17896	G	GG	AAAA
rs1488337	A_20_P00201129	chrX	121102142	178875	NN	AA	AAGG
rs12850095	A_20_P00201130	chrX	121103707	142662	C	NN	NN
rs1592092	A_20_P00303038	chrX	121165610	125658	G	GG	GGTT
rs5957884	A_20_P00201133	chrX	121238054	161085	T	TT	TTTT
rs5957895	A_20_P00201134	chrX	121270661	52172	T	TT	TTTT
rs12859125	A_20_P00201136	chrX	121344633	22807	A	AA	GGGG
rs1166822	A_20_P00201137	chrX	121381801	34520	G	AG	GGGG
rs5957970	A_20_P00201138	chrX	121414291	121558	NN	NN	NN
rs1481181	A_20_P00303045	chrX	121445429	150363	C	CC	CCCC
rs2448851	A_20_P00201140	chrX	121468185	79641	NN	CC	CCCC
rs5956478	A_20_P00201145	chrX	121634132	98134	NN	NN	NN
rs7883110	A_20_P00303052	chrX	121805775	152706	G	NN	NN
rs12010774	A_20_P00303053	chrX	121812033	21903	T	TT	TTTT
rs16996922	A_20_P00201148	chrX	121853391	95739	A	AA	AAAA
rs5958109	A_20_P00201149	chrX	121947195	138126	G	GC	GGGG
rs4372129	A_20_P00201151	chrX	121966203	125015	T	TT	CCCC
rs5911443	A_20_P00201152	chrX	121990427	39089	NN	NN	NN
rs2213539	A_20_P00201154	chrX	122249169	162922	NN	NN	NN
rs6648957	A_20_P00303061	chrX	122279181	66286	NN	AA	CCAA
rs12012579	A_20_P00303063	chrX	122300038	54065	C	CC	NN
rs5911538	A_20_P00201158	chrX	122309029	169522	NN	TC	TTTT
rs2157292	A_20_P00201159	chrX	122354683	63410	NN	AA	GGGG
rs16997198	A_20_P00303066	chrX	122363802	28460	NN	NN	NN
rs5911557	A_20_P00303067	chrX	122366636	55566	A	AA	AAAA
rs4825845	A_20_P00201162	chrX	122380720	121829	T	TT	GGGG
rs7063309	A_20_P00201163	chrX	122384743	56818	C	CG	CCGG
rs2157271	A_20_P00303070	chrX	122393150	99675	NN	CT	TTTT
rs2097395	A_20_P00303071	chrX	122414696	11738	G	GG	AAAA
rs5958212	A_20_P00201166	chrX	122427614	105989	G	GG	GGAA
rs5909987	A_20_P00303073	chrX	122435932	70753	NN	TC	TTTT

SNP ID	Probe Name	Chromosome	SNP Position	Feature Number	Father	Mother	Proband
rs5911581	A_20_P00303074	chrX	122436118	34321	NN	AC	AAAA
rs5911612	A_20_P00303076	chrX	122513284	16310	C	CC	CCTT
rs5910002	A_20_P00201171	chrX	122515696	23981	A	GG	GGAA
rs16997349	A_20_P00303078	chrX	122534942	84176	C	CC	CCTT
rs2851733	A_20_P00201174	chrX	122591071	118572	C	NN	NN
rs4501712	A_20_P00201175	chrX	122652411	82499	C	CC	NN
rs2498046	A_20_P00303082	chrX	122722456	12152	T	TT	TTTT
rs2050002	A_20_P00201177	chrX	122907594	54378	A	AA	AAAA
rs5958318	A_20_P00303086	chrX	122992822	15874	NN	CC	CCCT
rs16999350	A_20_P00201181	chrX	123039939	149819	G	GG	GGGG
rs5956589	A_20_P00303089	chrX	123057336	161189	A	NN	NN
rs4027740	A_20_P00201184	chrX	123285773	120744	NN	NN	NN
rs7888229	A_20_P00303092	chrX	123322496	28671	G	AG	AAGG
rs5958424	A_20_P00201187	chrX	123350694	38652	C	NN	NN
rs983042	A_20_P00303096	chrX	123373673	138988	C	GG	CCCC
rs5958445	A_20_P00303097	chrX	123414023	63884	G	GG	CCCC
rs5956646	A_20_P00303098	chrX	123488238	67293	C	CG	CCCC
rs5911841	A_20_P00201194	chrX	123569434	81332	C	NN	CCCC
rs2283769	A_20_P00303101	chrX	123582309	121946	T	TT	CCCC
rs7879039	A_20_P00201197	chrX	123600988	26106	G	GG	NN
rs2283765	A_20_P00303104	chrX	123632392	49476	A	AG	AAAA
rs5956662	A_20_P00303106	chrX	123672135	91973	NN	NN	NN
rs2843518	A_20_P00201201	chrX	123688200	154312	NN	TT	TTTT
rs2843496	A_20_P00303108	chrX	123708664	137230	C	NN	NN
rs2858421	A_20_P00201203	chrX	123710815	51689	NN	AA	AAGG
rs34969356	A_20_P00303110	chrX	123866368	148498	C	CT	TTTT
rs7050956	A_20_P00201205	chrX	123984382	118896	C	CC	TTTT
rs17325561	A_20_P00303112	chrX	124118994	153462	NN	AA	AAGG
rs7881406	A_20_P00201207	chrX	124155965	127851	G	NN	GGGG
rs2157262	A_20_P00201209	chrX	124276130	155499	NN	TC	CCCC
rs10521731	A_20_P00201210	chrX	124297173	112638	C	CC	CCCC
rs16998359	A_20_P00303117	chrX	124307609	71384	A	AA	AAGG
rs5958707	A_20_P00201212	chrX	124342361	33059	T	TT	TTTT
rs6655850	A_20_P00201213	chrX	124416058	25256	C	CC	CCCC
rs3101160	A_20_P00303120	chrX	124531501	117050	NN	NN	NN
rs1993601	A_20_P00303121	chrX	124535285	131014	A	AA	GGGG
rs3101155	A_20_P00201216	chrX	124537838	65536	NN	NN	NN
rs1458961	A_20_P00303123	chrX	124611081	82448	G	GG	CCGG
rs2157148	A_20_P00201218	chrX	124712710	103849	NN	GG	CCCC
rs28473050	A_20_P00303125	chrX	124718412	140760	NN	NN	NN
rs2819540	A_20_P00201220	chrX	124749542	94021	T	TT	TTTT
rs5930358	A_20_P00201222	chrX	124874812	88656	G	GG	AAGG
rs17303204	A_20_P00201223	chrX	125001599	167013	C	CC	CCCC
rs437170	A_20_P00201224	chrX	125061187	116799	T	TT	TTTT
rs209632	A_20_P00201225	chrX	125127881	112298	NN	NN	AAAA
rs5930573	A_20_P00303134	chrX	125165220	179637	G	NN	NN
rs2186039	A_20_P00303138	chrX	125384433	109267	NN	NN	NN

SNP ID	Probe Name	Chromosome	SNP Position	Feature Number	Father	Mother	Proband
rs1290565	A_20_P00201234	chrX	125474225	1556	C	CC	CCCC
rs845320	A_20_P00303141	chrX	125488313	22109	NN	NN	NN
rs4829815	A_20_P00201236	chrX	125507148	1769	G	GG	GGGG
rs6635312	A_20_P00201237	chrX	125564627	39136	NN	NN	NN
rs5974670	A_20_P00201238	chrX	125625551	164980	A	GA	AAAA
rs845300	A_20_P00303145	chrX	125626712	133380	NN	TG	TTTT
rs4829952	A_20_P00303147	chrX	125795845	140539	A	AG	GGGG
rs5974806	A_20_P00303148	chrX	125815787	31342	A	AA	AAAA
rs11796631	A_20_P00201243	chrX	125820299	152299	NN	NN	NN
rs2235079	A_20_P00303151	chrX	125829687	39355	NN	TT	TTTT
rs2235077	A_20_P00303152	chrX	125829805	152149	NN	CC	CCCC
rs5931680	A_20_P00201247	chrX	125860970	180492	NN	GG	AAAA
rs10081894	A_20_P00201248	chrX	126007226	112302	G	NN	NN
rs5930049	A_20_P00201249	chrX	126053341	10892	C	NN	NN
rs7892273	A_20_P00303156	chrX	126080235	9564	C	TT	CCCC
rs5931806	A_20_P00303157	chrX	126137484	145073	C	CC	NN
rs5930059	A_20_P00201252	chrX	126157448	129513	G	NN	NN
rs204329	A_20_P00201253	chrX	126160797	59312	NN	GG	CCGG
rs200726	A_20_P00201255	chrX	126453449	55902	T	TT	TTCC
rs2843474	A_20_P00201256	chrX	126512946	180658	C	CC	CCCC
rs3920517	A_20_P00201259	chrX	126636336	34501	C	CC	NN
rs4338242	A_20_P00303167	chrX	126776626	156202	C	CC	AACC
rs5930167	A_20_P00303168	chrX	126836427	172106	A	NN	NN
rs2001055	A_20_P00303169	chrX	126978449	87955	G	GG	TTTT
rs226552	A_20_P00201264	chrX	126990817	66536	G	GG	GGGG
rs4830061	A_20_P00201267	chrX	127108810	176202	G	GG	AAAA
rs803238	A_20_P00201269	chrX	127169420	105216	T	NN	TTTT
rs670607	A_20_P00303177	chrX	127272701	61585	C	CC	NN
rs240194	A_20_P00201273	chrX	127441816	172579	NN	AG	AAAA
rs7887222	A_20_P00303180	chrX	127490442	95173	C	CC	GGGG
rs16997677	A_20_P00201276	chrX	127593433	163602	T	TT	TTTT
rs2066968	A_20_P00303183	chrX	127611858	37857	G	GG	GGGG
rs5930261	A_20_P00201278	chrX	127625056	162367	NN	GG	GGGG
rs17313456	A_20_P00201279	chrX	127724972	8746	C	CC	TTTT
rs5932471	A_20_P00303186	chrX	127774845	138977	G	NN	NN
rs10218225	A_20_P00303187	chrX	127785423	145429	G	GG	AAAA
rs6637459	A_20_P00201283	chrX	127796993	10027	C	TC	TTTT
rs16315	A_20_P00201284	chrX	127834518	122003	C	NN	NN
rs5932511	A_20_P00303191	chrX	127944087	48202	G	GG	CCCC
rs4416970	A_20_P00201286	chrX	127948120	81654	G	GG	GGGG
rs6529307	A_20_P00303195	chrX	127989186	136149	G	NN	NN
rs2858157	A_20_P00303196	chrX	127998076	24236	NN	NN	CCCC
rs2746451	A_20_P00303197	chrX	128024866	139259	NN	NN	NN
rs16999885	A_20_P00303198	chrX	128049432	1911	T	TT	NN
rs2746449	A_20_P00201293	chrX	128068080	86564	NN	NN	NN
rs2746448	A_20_P00303200	chrX	128068387	28713	C	NN	NN
rs16992739	A_20_P00201295	chrX	128082290	44400	A	AA	AATT

SNP ID	Probe Name	Chromosome	SNP Position	Feature Number	Father	Mother	Proband
rs1417946	A_20_P00303203	chrX	128133911	170248	G	NN	GGGG
rs12390193	A_20_P00201298	chrX	128186895	59300	T	TT	TTTT
rs7882525	A_20_P00201300	chrX	128245982	113265	NN	GG	AAAA
rs5976996	A_20_P00201301	chrX	128310857	55948	C	NN	NN
rs2885947	A_20_P00201305	chrX	128550791	92410	C	CC	TTTT
rs2072837	A_20_P00201308	chrX	128690832	21173	A	GA	GGGG
rs2239747	A_20_P00201309	chrX	128708956	151289	NN	NN	NN
rs5977139	A_20_P00201310	chrX	128817632	55296	G	NN	NN
rs12558019	A_20_P00201311	chrX	128823071	173288	T	CC	TTTT
rs5930356	A_20_P00201313	chrX	128842121	74089	T	NN	NN
rs12014323	A_20_P00201319	chrX	129272520	77006	NN	NN	CCCC
rs5932749	A_20_P00201323	chrX	129487952	3597	T	TC	TTCC
rs5932754	A_20_P00201324	chrX	129515071	121146	T	GG	TTTT
rs5975194	A_20_P00201325	chrX	129576014	9298	NN	NN	CCCC
rs6634785	A_20_P00303232	chrX	129605707	139682	A	CC	AAAA
rs5977330	A_20_P00303234	chrX	129768608	17261	G	GG	AAAA
rs5930426	A_20_P00303236	chrX	129962264	36474	G	NN	NN
rs3859991	A_20_P00201333	chrX	130222535	107458	NN	GG	AAAA
rs7058605	A_20_P00303240	chrX	130233598	65678	NN	GG	AAGG
rs12389660	A_20_P00303241	chrX	130286046	128739	T	NN	NN
rs5932855	A_20_P00201336	chrX	130300180	176947	NN	CC	CCCC
rs17000821	A_20_P00201338	chrX	130324329	22630	NN	TT	TTGG
rs4073307	A_20_P00303245	chrX	130422622	132133	NN	GG	NN
rs6529475	A_20_P00201340	chrX	130431319	151868	G	GG	NN
rs7889515	A_20_P00303247	chrX	130431733	136175	G	AA	GGGG
rs17000365	A_20_P00201343	chrX	130450360	105889	T	TT	CCCC
rs41476153	A_20_P00303250	chrX	130460051	12731	G	GG	GGGG
rs17000392	A_20_P00201345	chrX	130465380	45768	G	GG	GGGG
rs7886509	A_20_P00201347	chrX	130582251	37403	T	TT	TTTT
rs2207164	A_20_P00201348	chrX	130593900	21037	NN	TT	NN
rs4829729	A_20_P00201349	chrX	130720671	98376	T	TT	TTTT
rs16992916	A_20_P00201351	chrX	130826305	62978	NN	CC	CCCC
rs202729	A_20_P00201354	chrX	131034790	84706	G	AG	AAAA
rs17000937	A_20_P00303262	chrX	131236984	125669	A	AA	GGGG
rs5933118	A_20_P00201357	chrX	131321571	109802	A	NN	NN
rs5977695	A_20_P00303266	chrX	131528839	61488	C	CC	AAAA
rs11798681	A_20_P00303267	chrX	131764393	125575	NN	TT	TTTT
rs1034424	A_20_P00303269	chrX	131984559	51093	T	CC	TTTT
rs5977775	A_20_P00201364	chrX	132022987	175070	T	AA	AAAA
rs4830252	A_20_P00303271	chrX	132027297	39143	C	CC	NN
rs6638037	A_20_P00201366	chrX	132049113	163869	A	GG	AAAA
rs17000367	A_20_P00201367	chrX	132057464	73109	A	AA	AATT
rs1018794	A_20_P00303275	chrX	132127845	177859	T	CC	TTTT
rs5933260	A_20_P00201371	chrX	132163703	121427	C	CT	TTTT
rs5977849	A_20_P00201372	chrX	132409641	170619	NN	NN	NN
rs11096365	A_20_P00201373	chrX	132450974	166673	NN	TT	NN
rs12010529	A_20_P00201374	chrX	132503487	5865	C	NN	CCCC

SNP ID	Probe Name	Chromosome	SNP Position	Feature Number	Father	Mother	Proband
rs12011132	A_20_P00303282	chrX	132534757	18369	NN	NN	NN
rs7055943	A_20_P00303284	chrX	132831297	109912	T	NN	NN
rs1003856	A_20_P00201379	chrX	132988656	32894	C	TT	TTTT
rs5975436	A_20_P00201381	chrX	133156268	122705	G	NN	NN
rs2142010	A_20_P00201382	chrX	133247561	155682	T	TT	CCCC
rs12394384	A_20_P00201383	chrX	133330216	30420	C	CC	CCCC
rs4830283	A_20_P00303290	chrX	133451057	79951	T	GG	GGGG
rs5933404	A_20_P00201386	chrX	133498300	139735	G	AG	GGGG
rs4830290	A_20_P00303293	chrX	133647428	52809	NN	NN	NN
rs5978037	A_20_P00201388	chrX	133860424	78822	G	NN	NN
rs5933472	A_20_P00201390	chrX	134073029	97739	G	GG	TTTT
rs5933532	A_20_P00201393	chrX	134431222	20897	G	GG	GGGG
rs2159263	A_20_P00201394	chrX	134505523	176075	T	TT	TTAA
rs6633736	A_20_P00303301	chrX	134552166	175396	G	GG	AAAA
rs4240104	A_20_P00303302	chrX	134563778	89174	T	NN	NN
rs5930743	A_20_P00303305	chrX	134719047	39184	G	GG	GGGG
rs1937731	A_20_P00201403	chrX	134840655	68689	T	NN	NN
rs5975627	A_20_P00201405	chrX	134972878	163942	G	GG	AAAA
rs2501475	A_20_P00303312	chrX	134973369	74941	C	CC	GGGG
rs4829587	A_20_P00201407	chrX	135002573	30582	NN	NN	GGGG
rs7882434	A_20_P00303314	chrX	135015043	92681	C	CC	AAAA
rs6654311	A_20_P00201409	chrX	135108163	15021	NN	GG	NN
rs10521848	A_20_P00303316	chrX	135112228	126780	C	TT	CCCC
rs1994878	A_20_P00303317	chrX	135154918	31109	G	NN	NN
rs17001831	A_20_P00201412	chrX	135267779	31022	A	AA	TTTT
rs17001875	A_20_P00201413	chrX	135273179	109614	A	AA	AAAA
rs1089820	A_20_P00303322	chrX	135465929	37747	C	TT	CCCC
rs6528362	A_20_P00303323	chrX	135473096	97727	A	AA	AAAA
rs1360162	A_20_P00201418	chrX	135474929	131469	G	GG	CCCC
rs2183252	A_20_P00201420	chrX	135532954	103439	NN	NN	NN
rs1203583	A_20_P00303327	chrX	135534948	110113	T	TT	CCCC
rs3027841	A_20_P00303329	chrX	135585164	144416	C	CC	TTTT
rs3027867	A_20_P00303330	chrX	135631972	111052	A	AA	AAAA
rs2518886	A_20_P00201425	chrX	135670593	123198	C	CC	NN
rs2518894	A_20_P00303332	chrX	135677710	172661	NN	TT	TTTT
rs2518903	A_20_P00303333	chrX	135681110	47588	NN	TT	TTTT
rs175735	A_20_P00201428	chrX	135682327	104589	NN	TT	TTTT
rs5974624	A_20_P00201430	chrX	135830691	100872	A	NN	NN
rs5930994	A_20_P00201432	chrX	135876353	55118	NN	NN	NN
rs5931006	A_20_P00303339	chrX	135896560	49497	A	AA	GGGG
rs5975811	A_20_P00201434	chrX	135950219	122596	C	CC	CCCC
rs10482345	A_20_P00303341	chrX	135989221	69500	NN	NN	NN
rs7058370	A_20_P00201436	chrX	136119563	72685	C	CC	NN
rs2787128	A_20_P00201439	chrX	136200578	13017	G	GG	GGGG
rs5975888	A_20_P00201440	chrX	136336712	138162	G	AA	GGGG
rs5929811	A_20_P00303347	chrX	136356727	80755	G	TT	GGGG
rs16993403	A_20_P00303348	chrX	136426096	56190	G	GG	GGGG

SNP ID	Probe Name	Chromosome	SNP Position	Feature Number	Father	Mother	Proband
rs17001430	A_20_P00201444	chrX	136470643	171137	A	CC	AAAA
rs1206238	A_20_P00201445	chrX	136496830	155462	C	NN	NN
rs1206215	A_20_P00303352	chrX	136534217	68597	T	TT	TTTT
rs4829886	A_20_P00201447	chrX	136576926	44015	NN	NN	NN
rs2859253	A_20_P00201448	chrX	136597585	115484	NN	NN	NN
rs6635446	A_20_P00303355	chrX	136600502	143066	NN	NN	NN
rs2746112	A_20_P00303356	chrX	136613220	2771	T	CC	TTTT
rs2366298	A_20_P00303358	chrX	136727435	68882	NN	NN	NN
rs12013075	A_20_P00303360	chrX	136870786	46994	NN	CC	CCCC
rs5976027	A_20_P00201456	chrX	137074124	1975	NN	GG	GGGG
rs5931302	A_20_P00303363	chrX	137154908	48193	C	GG	GGGG
rs6633979	A_20_P00201459	chrX	137312885	37951	A	CC	CCCC
rs5974738	A_20_P00201464	chrX	137466260	53437	T	TT	TTTT
rs2211286	A_20_P00201465	chrX	137468464	100366	NN	NN	NN
rs5931421	A_20_P00201466	chrX	137508689	179274	G	GG	GGGG
rs5931430	A_20_P00303373	chrX	137533231	88544	NN	NN	NN
rs7063777	A_20_P00201468	chrX	137546094	109779	C	NN	NN
rs5931457	A_20_P00303376	chrX	137602664	144260	A	AA	NN
rs5976166	A_20_P00303378	chrX	137634373	161621	G	GG	GGGG
rs5929946	A_20_P00303380	chrX	137832884	27239	T	TG	GGGG
rs7056214	A_20_P00201475	chrX	137988662	60981	C	CC	CCCC
rs7059725	A_20_P00201476	chrX	137988737	137860	NN	NN	NN
rs17001838	A_20_P00201477	chrX	137993106	31531	T	TT	CCCC
rs475819	A_20_P00201478	chrX	138021436	93430	G	NN	NN
rs4829636	A_20_P00303385	chrX	138031265	74422	NN	GG	NN
rs6635751	A_20_P00201480	chrX	138074362	152233	T	CC	TTTT
rs17331422	A_20_P00303389	chrX	138187934	96983	A	AA	AAAA
rs5931582	A_20_P00303392	chrX	138263904	24653	A	AA	AAAA
rs4829964	A_20_P00201487	chrX	138267771	97016	NN	NN	NN
rs6635800	A_20_P00303396	chrX	138390890	71457	A	AA	TTTT
rs5931610	A_20_P00201491	chrX	138415619	3696	NN	AC	NN
rs2223690	A_20_P00201494	chrX	138447195	126481	NN	CG	CCCC
rs5931656	A_20_P00303402	chrX	138576517	102599	C	CC	CCTT
rs11796672	A_20_P00303403	chrX	138587625	163105	T	TT	TTTT
rs4149749	A_20_P00201498	chrX	138643225	37281	C	NN	NN
rs5954579	A_20_P00201500	chrX	138879662	43933	C	CC	NN
rs6635968	A_20_P00303410	chrX	139136708	130990	C	GC	GGGG
rs5953968	A_20_P00303411	chrX	139189102	140590	C	CC	CCCC
rs5955392	A_20_P00303412	chrX	139201606	146782	C	CC	CCCC
rs77742	A_20_P00201510	chrX	139514602	76064	A	AA	AAAA
rs203652	A_20_P00201513	chrX	139627530	86664	C	CC	TTTT
rs6634184	A_20_P00303420	chrX	139732666	51938	NN	AA	AAAA
rs6636116	A_20_P00201515	chrX	139865162	110170	G	NN	NN
rs5907056	A_20_P00201516	chrX	139874378	154376	T	TC	TTCC
rs5907697	A_20_P00303426	chrX	139918162	13901	C	TT	TTCC
rs5907077	A_20_P00303427	chrX	139923111	106936	C	CC	GGGG
rs7885192	A_20_P00201524	chrX	139937398	34828	T	TT	TTTT

SNP ID	Probe Name	Chromosome	SNP Position	Feature Number	Father	Mother	Proband
rs5907079	A_20_P00303431	chrX	139940457	125704	C	NN	NN
rs4824967	A_20_P00201527	chrX	139961990	100532	C	NN	NN
rs5953580	A_20_P00201531	chrX	140354434	70547	C	TC	TTCC
rs845163	A_20_P00303438	chrX	140371091	175922	G	AG	AAAA
rs844946	A_20_P00303439	chrX	140453748	153758	NN	NN	NN
rs12836845	A_20_P00201537	chrX	140724340	150813	C	CT	CCCC
rs7891103	A_20_P00201538	chrX	140814831	88016	NN	NN	NN
rs34945378	A_20_P00303446	chrX	140879702	64453	T	TT	TTTT
rs5953634	A_20_P00201542	chrX	140961960	94320	NN	NN	NN
rs4825057	A_20_P00201545	chrX	141120176	66737	C	CC	TTTT
rs16979988	A_20_P00201546	chrX	141156616	69893	T	NN	NN
rs6528962	A_20_P00201547	chrX	141195385	137326	NN	NN	NN
rs6528963	A_20_P00201548	chrX	141195450	171848	A	AG	AAGG
rs7065189	A_20_P00303455	chrX	141234260	20693	C	CC	CCCC
rs10482384	A_20_P00201551	chrX	141305556	18599	G	GG	GGGG
rs2223456	A_20_P00303460	chrX	141323367	83689	G	NN	NN
rs16978761	A_20_P00201555	chrX	141347922	179265	A	AA	GGGG
rs5908241	A_20_P00303462	chrX	141354506	28853	NN	NN	NN
rs5954599	A_20_P00303463	chrX	141365278	149027	G	GG	GGGG
rs16978791	A_20_P00201558	chrX	141367460	77557	NN	NN	NN
rs6636673	A_20_P00201559	chrX	141503758	7533	C	NN	NN
rs5908318	A_20_P00201561	chrX	141525534	11465	G	GG	GGAA
rs5908325	A_20_P00303468	chrX	141563340	108845	G	GG	GGAA
rs5953740	A_20_P00303469	chrX	141670437	95850	C	TC	TTCC
rs2103844	A_20_P00303470	chrX	141689825	65249	G	GG	GGGG
rs41341244	A_20_P00201565	chrX	141690336	93126	G	GG	GGGG
rs5954800	A_20_P00303473	chrX	141747996	65000	A	AA	GGGG
rs13440607	A_20_P00303474	chrX	141750938	167278	G	GG	GGGG
rs5908388	A_20_P00201569	chrX	141769928	145790	NN	NN	CCCC
rs4539002	A_20_P00303476	chrX	141783952	29604	NN	NN	TTTT
rs11096013	A_20_P00303477	chrX	141786770	180261	T	TT	TTGG
rs12688989	A_20_P00201572	chrX	141789677	64746	NN	NN	NN
rs12008814	A_20_P00303479	chrX	141790302	132898	T	TT	TTTT
rs6529056	A_20_P00201574	chrX	141837772	65105	A	AA	AAAA
rs12009282	A_20_P00201575	chrX	141873409	176986	T	TT	TTTT
rs16979046	A_20_P00201577	chrX	141913279	150357	A	AA	AAAA
rs7056670	A_20_P00303484	chrX	141916934	139106	NN	TT	TTTT
rs12833105	A_20_P00201583	chrX	142107408	162073	NN	GG	GGGG
rs11798366	A_20_P00201584	chrX	142126964	111163	A	AA	AAGG
rs5908568	A_20_P00201585	chrX	142239618	154036	NN	NN	NN
rs2235085	A_20_P00201587	chrX	142283962	67937	NN	AA	AAAA
rs5953815	A_20_P00201588	chrX	142287357	146489	T	TT	TTTT
rs5954991	A_20_P00201589	chrX	142349193	100850	NN	AA	AAAA
rs5907417	A_20_P00303498	chrX	142425333	64364	NN	CC	NN
rs5908645	A_20_P00201593	chrX	142433590	93925	C	CC	NN
rs7886052	A_20_P00201595	chrX	142534369	110977	G	NN	NN
rs5955024	A_20_P00201596	chrX	142542239	124965	NN	NN	CCCC

SNP ID	Probe Name	Chromosome	SNP Position	Feature Number	Father	Mother	Proband
rs5953849	A_20_P00303505	chrX	142593003	79308	G	NN	NN
rs6637027	A_20_P00201602	chrX	142656443	94346	C	CC	CCCC
rs12391812	A_20_P00201603	chrX	142661550	104922	A	AG	AAAA
rs5908713	A_20_P00303510	chrX	142677151	123213	NN	TT	NN
rs2207582	A_20_P00201608	chrX	142737999	156815	NN	TC	TTTT
rs878882	A_20_P00201611	chrX	142790988	24975	T	NN	NN
rs5907474	A_20_P00303518	chrX	142817807	140842	C	CC	NN
rs5955174	A_20_P00201616	chrX	143036780	25611	C	CC	CCCC
rs859949	A_20_P00201621	chrX	143363119	54702	G	GG	AAAA
rs9780436	A_20_P00201622	chrX	143374708	101212	C	NN	NN
rs6649725	A_20_P00201624	chrX	143393210	69274	G	GG	GGGG
rs5955337	A_20_P00303531	chrX	143458688	89453	NN	NN	CCCC
rs11096121	A_20_P00303532	chrX	143476289	33668	NN	NN	NN
rs2815681	A_20_P00303537	chrX	143734156	58898	A	AA	AAAA
rs12013868	A_20_P00303539	chrX	143736145	101265	G	GG	GGGG
rs11094371	A_20_P00201636	chrX	143828652	98839	G	GG	AAAA
rs5966111	A_20_P00303545	chrX	143891490	42020	T	NN	NN
rs7889172	A_20_P00201642	chrX	143939707	84261	T	AA	AAAA
rs10482174	A_20_P00303549	chrX	143944567	59091	NN	TT	TTTT
rs7885767	A_20_P00201644	chrX	143994475	159781	NN	TT	CCTT
rs5966402	A_20_P00201645	chrX	144064675	133013	T	TT	NN
rs914820	A_20_P00303554	chrX	144138256	93545	NN	NN	NN
rs5966434	A_20_P00201649	chrX	144219076	46977	T	CT	CCTT
rs5920463	A_20_P00303556	chrX	144299618	153119	NN	GG	GGGA
rs5965839	A_20_P00201651	chrX	144331367	84822	NN	NN	NN
rs16995439	A_20_P00303561	chrX	144543325	74589	G	NN	GGGG
rs5965860	A_20_P00201657	chrX	144579741	19569	T	TC	CCCC
rs1174179	A_20_P00303564	chrX	144609117	170389	A	AA	AAAA
rs5920565	A_20_P00201659	chrX	144614280	138164	NN	NN	NN
rs16993173	A_20_P00303566	chrX	144662441	17217	A	AA	AAAA
rs1143856	A_20_P00201662	chrX	144690368	25989	A	AC	CCCC
rs5965630	A_20_P00201663	chrX	144745170	88457	C	CC	CCCC
rs2842670	A_20_P00201665	chrX	144816043	111400	G	AA	GGGG
rs5919896	A_20_P00303574	chrX	144858373	133526	G	CC	GGGG
rs5919909	A_20_P00303575	chrX	144910142	21073	G	GG	GGGG
rs5920020	A_20_P00303580	chrX	145232687	17010	NN	TT	TTTT
rs16993581	A_20_P00201675	chrX	145280008	176228	T	TT	TTCC
rs5919668	A_20_P00303584	chrX	145436429	91093	C	NN	NN
rs7062770	A_20_P00201679	chrX	145505010	142414	C	CC	CCCC
rs232688	A_20_P00201680	chrX	145505768	137699	A	AA	AAGG
rs12007117	A_20_P00201681	chrX	145570278	90482	A	NN	NN
rs5920171	A_20_P00303588	chrX	145586725	26592	T	NN	NN
rs5966223	A_20_P00201683	chrX	145591175	176203	T	TT	TTTT
rs2385657	A_20_P00201685	chrX	145631205	53729	G	GG	AAAA
rs6626671	A_20_P00201686	chrX	145712494	141864	G	GG	GGAA
rs10218124	A_20_P00303594	chrX	145838032	74058	A	AA	CCCC
rs5919728	A_20_P00303596	chrX	145848770	120088	NN	AA	CCCC

SNP ID	Probe Name	Chromosome	SNP Position	Feature Number	Father	Mother	Proband
rs6525744	A_20_P00201693	chrX	145911930	82394	T	NN	TTTT
rs5951897	A_20_P00201694	chrX	145938805	44136	C	NN	NN
rs1336834	A_20_P00201695	chrX	145939843	6506	T	TC	CCCC
rs12010752	A_20_P00201697	chrX	145964872	107068	NN	NN	NN
rs1336840	A_20_P00303604	chrX	145991136	160191	NN	CT	CCCC
rs1981490	A_20_P00201699	chrX	145991725	86417	NN	TT	TTTT
rs6626802	A_20_P00303606	chrX	146002069	118374	NN	AG	AAAA
rs5904904	A_20_P00303610	chrX	146081786	4103	NN	NN	NN
rs16994008	A_20_P00201705	chrX	146121647	123505	T	TC	TTTT
rs5951923	A_20_P00303612	chrX	146156542	122923	G	NN	NN
rs5904984	A_20_P00303613	chrX	146268151	147042	NN	TT	TTTT
rs7885592	A_20_P00303615	chrX	146330007	46599	G	GG	GGGG
rs5905021	A_20_P00303616	chrX	146335339	74497	T	TT	TTTT
rs5905022	A_20_P00303617	chrX	146335634	143226	A	AA	CCCC
rs16994084	A_20_P00201712	chrX	146403304	123693	G	GG	GGGG
rs16994087	A_20_P00303619	chrX	146447343	99646	C	NN	NN
rs16994104	A_20_P00303620	chrX	146464912	172587	C	NN	NN
rs6626889	A_20_P00303621	chrX	146528063	141334	G	GG	NN
rs2761619	A_20_P00303624	chrX	146723480	155903	NN	NN	NN
rs5952033	A_20_P00201721	chrX	146758895	59561	T	TT	TTTT
rs5904778	A_20_P00201723	chrX	146899835	113597	T	NN	NN
rs12011072	A_20_P00201724	chrX	146900547	1508	T	TT	TTGG
rs2197710	A_20_P00201726	chrX	146975441	155345	C	GG	GGCC
rs5904843	A_20_P00201728	chrX	147106925	3848	A	AA	AACC
rs6525877	A_20_P00303636	chrX	147158718	19464	C	CC	TTCC
rs4266775	A_20_P00201732	chrX	147244696	122162	G	GG	AAGG
rs6540401	A_20_P00201733	chrX	147318813	130647	C	CC	NN
rs5936467	A_20_P00201734	chrX	147346449	55679	A	AA	AAAA
rs2191949	A_20_P00201735	chrX	147370355	105491	G	GG	GGGG
rs5980510	A_20_P00201738	chrX	147475722	97239	C	CC	CCCC
rs5980515	A_20_P00201739	chrX	147502461	38336	NN	TT	TTTT
rs5980538	A_20_P00303647	chrX	147581470	74224	C	TC	TTTT
rs718797	A_20_P00303649	chrX	147666486	119106	G	NN	NN
rs4844064	A_20_P00201744	chrX	147680647	132742	A	NN	NN
rs241109	A_20_P00303651	chrX	147744465	6767	C	CC	CCCC
rs6641463	A_20_P00303653	chrX	147864249	42586	A	GG	GGGG
rs16994733	A_20_P00303654	chrX	147890895	88546	A	AA	AAAA
rs12008688	A_20_P00201750	chrX	147997440	3890	NN	GC	CCCC
rs6641476	A_20_P00303657	chrX	148027547	170115	C	CC	TTTT
rs16994865	A_20_P00303658	chrX	148035905	42647	T	TT	TTTT
rs5980647	A_20_P00201753	chrX	148207599	56479	NN	NN	NN
rs3135484	A_20_P00303660	chrX	148210200	31835	G	GG	GGGG
rs10521879	A_20_P00201755	chrX	148301414	100908	NN	GG	NN
rs7061632	A_20_P00303662	chrX	148306884	147397	C	NN	NN
rs5936246	A_20_P00303663	chrX	148337849	155553	G	GG	CCCC
rs1882726	A_20_P00303664	chrX	148343411	163561	NN	NN	NN
rs5936253	A_20_P00303665	chrX	148358734	147584	NN	TT	TTTT

SNP ID	Probe Name	Chromosome	SNP Position	Feature Number	Father	Mother	Proband
rs10521884	A_20_P00201765	chrX	148542836	116036	NN	NN	NN
rs5936287	A_20_P00303672	chrX	148557940	174737	C	CC	CCCC
rs454992	A_20_P00303675	chrX	148885435	38918	NN	NN	NN
rs6649478	A_20_P00303678	chrX	149265386	146729	C	CC	NN
rs9779328	A_20_P00303679	chrX	149298673	15974	T	TT	TTCC
rs11094529	A_20_P00303680	chrX	149320212	66687	C	CC	CCCC
rs10776287	A_20_P00303681	chrX	149321090	45068	G	GG	NN
rs9697803	A_20_P00303682	chrX	149326556	101617	T	TT	TTTT
rs12687611	A_20_P00201782	chrX	149494254	148951	NN	CC	TTTT
rs12557374	A_20_P00201783	chrX	149494602	69303	C	CC	TTCC
rs5970497	A_20_P00303690	chrX	149517397	175857	NN	GG	NN
rs686328	A_20_P00201785	chrX	149542406	74534	NN	NN	NN
rs12008806	A_20_P00201786	chrX	149578110	167131	G	GG	GGGG
rs2266828	A_20_P00201787	chrX	149624093	6840	NN	NN	NN
rs2266835	A_20_P00303694	chrX	149652475	126624	T	TC	TTTT
rs12559341	A_20_P00201789	chrX	149676820	5569	C	GG	GGGG
rs4828708	A_20_P00201791	chrX	149688929	141001	G	CG	GGGG
rs1889131	A_20_P00201792	chrX	149700436	121328	G	GG	GGGG
rs222387	A_20_P00201794	chrX	149798354	28262	A	AA	AAAA
rs5925401	A_20_P00201795	chrX	149824237	55035	T	CT	CCCC
rs222415	A_20_P00303702	chrX	149834436	8766	T	TT	NN
rs6627324	A_20_P00201797	chrX	149881852	20270	C	TC	TTTT
rs1882710	A_20_P00201798	chrX	149894356	29264	A	AA	GGGG
rs12011604	A_20_P00201799	chrX	149965694	168792	C	TT	CCCC
rs11796789	A_20_P00303706	chrX	150040336	154370	NN	NN	NN
rs6627835	A_20_P00303707	chrX	150171247	115525	C	CC	NN
rs1202873	A_20_P00201804	chrX	150347755	141625	A	AA	AAAA
rs6627154	A_20_P00303712	chrX	150493100	60394	G	GG	AAGG
rs2369717	A_20_P00201807	chrX	150544915	41308	T	CT	CCCC
rs5924947	A_20_P00303714	chrX	150613245	56930	NN	TT	TTCC
rs5969793	A_20_P00201809	chrX	150627337	55	G	GG	GGGG
rs16995984	A_20_P00201810	chrX	150658010	89968	NN	NN	NN
rs12008463	A_20_P00201813	chrX	150668681	150744	A	AA	AAAA
rs12859750	A_20_P00201814	chrX	150810059	112479	G	NN	NN
rs5924996	A_20_P00303721	chrX	150847120	113783	G	GG	NN
rs4828637	A_20_P00201817	chrX	150849796	122791	NN	NN	NN
rs7054971	A_20_P00303724	chrX	150902802	126833	T	TT	TTTT
rs12686972	A_20_P00201819	chrX	150943361	123099	A	AA	CCAA
rs6627473	A_20_P00201820	chrX	150968662	125532	T	NN	NN
rs6526038	A_20_P00201821	chrX	150978737	83149	C	NN	NN
rs16996188	A_20_P00201822	chrX	151038186	60238	A	AA	AACC
rs5924702	A_20_P00201826	chrX	151090892	5745	C	AA	AAAA
rs5925082	A_20_P00201827	chrX	151157535	144358	G	GG	NN
rs2472367	A_20_P00201828	chrX	151187946	50643	G	GG	NN
rs2472368	A_20_P00303735	chrX	151189463	166205	A	AA	AAAA
rs5970194	A_20_P00201831	chrX	151240615	115529	G	GG	GGGG
rs6627526	A_20_P00201833	chrX	151252478	56064	NN	NN	GGGG

SNP ID	Probe Name	Chromosome	SNP Position	Feature Number	Father	Mother	Proband
rs5970269	A_20_P00201837	chrX	151482743	20247	NN	NN	NN
rs5970283	A_20_P00201838	chrX	151514635	142890	NN	NN	CCCC
rs7062928	A_20_P00303746	chrX	151627495	131437	A	AA	GGGG
rs17326876	A_20_P00201842	chrX	151673048	111443	NN	NN	NN
rs17174274	A_20_P00303752	chrX	152012986	135607	C	CC	CCTT
rs2071256	A_20_P00303753	chrX	152036452	70028	NN	GG	GGGG
rs36042591	A_20_P00303757	chrX	152226556	18612	G	NN	NN
rs4828757	A_20_P00303761	chrX	152492059	133607	NN	NN	NN
rs1894354	A_20_P00201861	chrX	152605526	163315	C	TC	TTTT
rs4898421	A_20_P00303771	chrX	152834160	103000	NN	GG	AAAA
rs5987129	A_20_P00303772	chrX	152944729	58460	G	NN	NN
rs2071132	A_20_P00303774	chrX	153216036	54607	A	GG	AAAA
rs11795678	A_20_P00201869	chrX	153265728	57608	G	AA	AAAA
rs5986958	A_20_P00303777	chrX	153390481	27172	G	GG	GGGG
rs2239466	A_20_P00201872	chrX	153554883	24681	NN	NN	NN
rs17091376	A_20_P00201873	chrX	153562458	114451	NN	NN	NN
rs762516	A_20_P00201877	chrX	153764663	111261	C	NN	NN
rs2048294	A_20_P00303784	chrX	154022646	169069	T	NN	NN
rs7881389	A_20_P00201879	chrX	154278824	74895	NN	AA	AAAA
rs523614	A_20_P00201881	chrX	154496166	144919	T	TC	NN
rs782310	A_20_P00201882	chrX	154819368	93219	NN	CC	NN
rs5940536	A_20_P00303789	chrX	154821956	167686	G	GG	NN
rs557132	A_20_P00201886	chrX	154929412	71775	NN	NN	NN
rs306873	A_20_P00303793	chrX	155011926	138633	TT	TT	CC
rs802488	A_20_P00201889	chrX	155045646	7087	NN	GG	GG
rs5983830	A_20_P00201891	chrX	155158531	145857	NN	GG	AA

Gray column indicates non-informative.

Blue and pink rows (columns) suggest paternal and maternal origins, respectively.
